# Functionalized Calixarenes as Promising Antibacterial Drugs to Face Antimicrobial Resistance

**DOI:** 10.3390/molecules28196954

**Published:** 2023-10-06

**Authors:** Maxime Mourer, Jean-Bernard Regnouf-de-Vains, Raphaël E. Duval

**Affiliations:** 1 Université de Lorraine, CNRS, L2CM, F-54000 Nancy, France; jean-bernard.regnouf@univ-lorraine.fr; 2 ABC Platform®, F-54505 Vandœuvre-lès-Nancy, France

**Keywords:** calixarenes, antibacterial drugs, bacterial infections, small molecules, functionalized calixarenes, antibacterial activity, nosocomial infections, antimicrobial resistance

## Abstract

Since the discovery of polyphenolic resins 150 years ago, the study of polymeric compounds named calix[*n*]arene has continued to progress, and those skilled in the art perfectly know now how to modulate this phenolic ring. Consequently, calix[*n*]arenes are now used in a large range of applications and notably in therapeutic fields. In particular, the calix[4]arene exhibits multiple possibilities for regioselective polyfunctionalization on both of its rims and offers researchers the possibility of precisely tuning the geometry of their structures. Thus, in the crucial research of new antibacterial active ingredients, the design of calixarenes finds its place perfectly. This review provides an overview of the work carried out in this aim towards the development of intrinsically active prodrogues or metallic calixarene complexes. Out of all the work of the community, there are some excellent activities emerging that could potentially place these original structures in a very good position for the development of new active ingredients.

## 1. Introduction/Context

The search for innovative treatments against infectious diseases is an important topical issue. With the emergence of new viral pathologies without any available treatment and the dissemination of antimicrobial resistance, both community- and hospital-acquired infections generate a major public health problem, the management of which represents a real challenge and a significant cost for society [1,2,3]. To deal with this situation, it is therefore crucial to develop new—if possible, innovative—anti-infective therapies [4,5]. Although the pathogens responsible for infectious diseases can be viruses or fungi, antibiotic-resistant infections are increasingly common; thus, bacterial resistance being, according to the WHO, are one of the “biggest threats to global health today” [6].

Bacterial resistance to antibiotics (i.e., natural resistance) is an old and natural phenomenon and is the basis of the spectrum of antibacterial compounds. However, the repeated or prolonged exposure of bacteria to the same active compound(s) most often results in the appearance of acquired resistance. This phenomenon, called selection (or selective) pressure, leads to the selection of resistant bacteria, and this resistance phenotype can be transmitted either horizontally or vertically to subsequent generations, depending on the nature of the mechanism of resistance [7,8]. The consequence is that, for several years, we have observed a dramatic increase (i) in the number of bacteria resistant to antibiotics, but also (ii) in the number of resistance mechanisms, which ultimately leads to the appearance and the spread of multidrug-resistant (MDR) bacteria. There is currently no evidence that these MDRs are more virulent than wild-type bacteria of the same species, but the small therapeutic arsenal remaining available can sometimes prove ineffective and cause great difficulties in patient care; this raises the risk of therapeutic impasse [9].

The extremely low number of new antibiotics developed and validated by the health authorities in recent years may also play an important role in the emergence of antimicrobial resistance; the “renewed interest” in colistin over the last ten years is the best example [10]. In September 2019, a total of 50 antibiotics (synthetic or biological (mono- or polyclonal antibodies)), combinations included, were in development, 32 of which target pathogenic organisms prioritized by the WHO. The WHO estimates that barely 10 of them could be on the market within the next 5 to 10 years. Unfortunately, only eight new antibacterial agents, with limited clinical benefits, have been approved since July 2017 [11]. These new treatments bring little to the existing situation and fail to cope with antimicrobial resistance [12].

In addition, the misuse of available antibiotics leads to a vicious circle which also contributes to the dissemination of both antimicrobial resistance and bacteria resistant to almost all available antibiotics all around the world. Parallelly, global antibiotic consumption increased by 65% between 2000 and 2015, with an estimated global consumption of 42.3 billion daily doses [13]. On this path, it is estimated that in 2030, overall consumption will be up to 200% higher than the 42 billion daily doses estimated in 2015.

Finally, it is quite easy to conclude that the fight against antibiotic resistance is a complex, multifactorial problem; however, at the same time, there is an urgent need to (re)act. Indeed, according to projections and if nothing is done, antibiotic resistance could cause 10 million deaths per year by 2050 [14]. If the “appropriate” use of antibiotics should allow us to save time, it has become clear that it is essential to develop new active ingredients, if possible with original mechanisms of action, in order to face antimicrobial resistance and bypass the current bacterial-resistance mechanisms.

For two decades, calixarenes have demonstrated remarkable activities in various biological fields—in particular, for the research and development of antimicrobial compounds such as antivirals, antibacterials, or antifungals. Indeed, the very wide possibilities for molecular design offered by the calixarenic platform place it in a good position for the development of new drugs with original modes of action.

In this review, we focus on the role of calixarenes in antibacterial applications and concentrate the bibliography only on discrete molecules, i.e., the largest molecular structures incorporating calixarenes are not considered (for example, polymers, dendrimers, metal nanoparticles, vesicles, etc.)

## 2. Objectives of this Review

Basket-type molecular architectures have found a place in the therapeutic field and more particularly in the development of active-ingredient-administration systems (increased solubility of active substances, stability, or bioavailability), for example, [15,16,17,18,19,20,21,22,23,24,25,26,27,28,29,30,31]. These systems are based on molecular recognition between the host macrocycle and the guest molecule forming a host-guest complex via non-covalent interactions. Among the best known of these macrocycles, capable of forming inclusion with various active molecules, are: the cyclodextrins, for example, [15,16,17]; the crown ethers, for example, [18,19,20]; the resorcinarenes, for example, [21,22,23]; the calix[*n*]arenes, for example, [24,25,26,27,28]; or the cucurbit[*n*]uriles, for example, [29,30,31].

Considered to be the third generation of host-supramolecules after the cyclodextrins and ether-crowns, the calix[4]arenes are becoming one of the most studied macromolecules, finding utility in a wide variety of fields, including: separative sciences, for example, [32,33,34]; drug delivery, for example, [35,36,37]; catalysis, for example, [38,39,40,41]; the nuclear industry, for example, [42,43,44]; sensor development, for example, [45,46,47]; or recovery of metals, for example, [48,49,50]. Moreover, the calix[4]arene derivatives take an important place in a large range of subjects associated with therapeutic fields, particularly due to their potential antibacterial properties. Thus, the growing number of studies dealing with the antibacterial properties of calix[*n*]arenes published in recent years has pushed us to propose the present review as a first assessment, which we hope is exhaustive.

## 3. Bibliographic Search

The bibliographic search was performed electronically using SciFinder, (CAPLUS and PUBMED database) as the search engine, until 2 September 2022. SciFinder search terms were as follows: “calix” AND (“bacterial” OR “microbial” OR “drugs”). The publication years of this subject matter ranged from 1955 to 2022.

## 4. Summary Description of the Structure of Calixarenes

**Figure 1:**
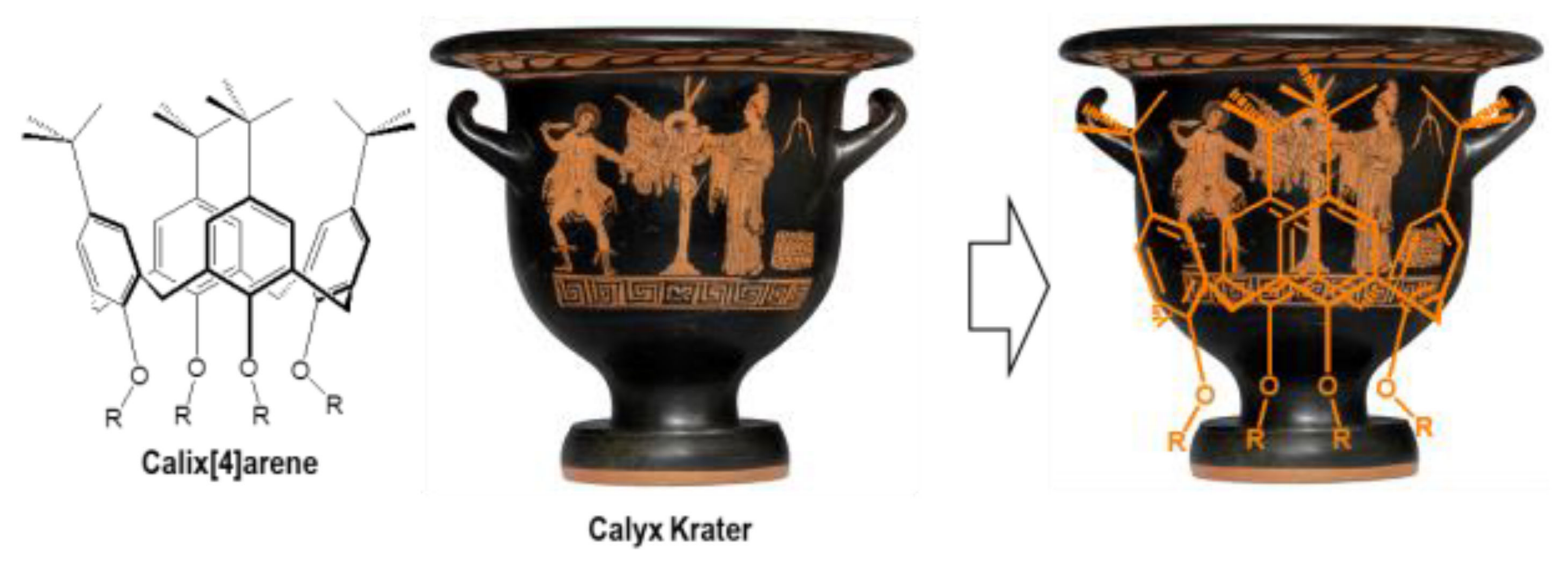
Analogy between calix[*n*]arene and a calyx krater.

This structure was discovered in the 19th century when A. von Baeyer [51,52] explored the base-catalysed condensation of phenol with formaldehyde. At the beginning of the 20th century, the commercialization of the phenolic resin “bakelite”, resulting from this type of condensation, started the studies on these polymeric compounds. The works of Niederl and Vogel in 1940 [53], and then Zinke and Ziegler in 1942 [54], allowed the proposition of a cyclic tetrameric structure, notably by their studies on condensation using para-substituted phenol. C. D. Gutsche confirmed, in the 1980s, the existence of cyclic oligomers of various sizes (4, 6, or 8 phenolic units). The denomination “calixarene” was used for the first time in 1978 by Gutsche [55]. This name was chosen for the similarity between this shape and a kind of Greek vase called a calyx krater; the suffix “arene” refers to the aromatic building blocks (Figure 1). Thus “calix[*n*]arene” corresponds to a macrocycle constituted of n phenolic units.

This name used initially by Gutsche for the macrocycles obtained by base-catalysed condensation of phenol with formaldehyde was then more widely used in a generic way for various cyclic structures (calixresorcinarene, thiacalixarene, azacalixarene…).

**Figure 2:**
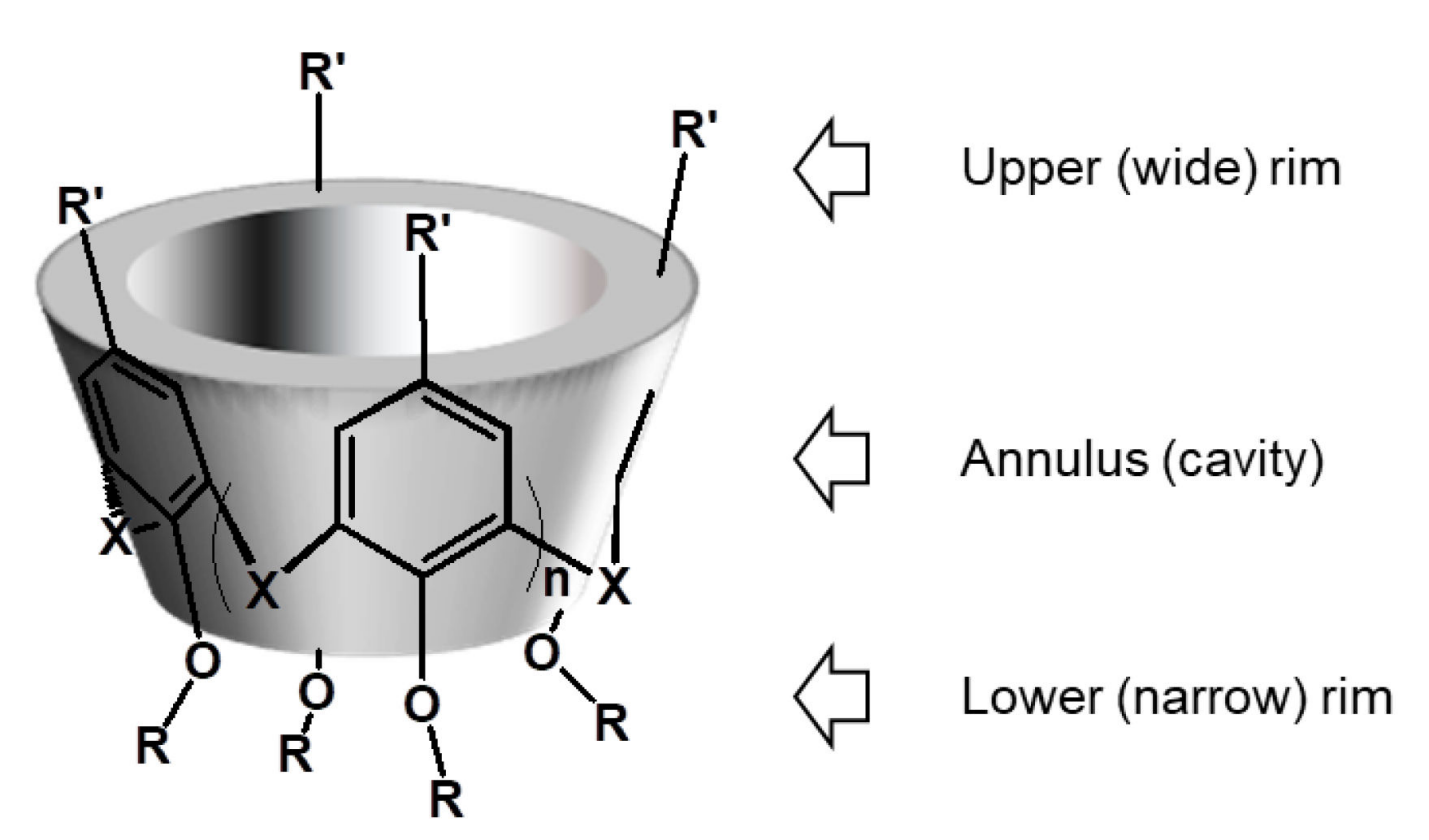
General structure of calix[*n*]arene. X = CH_2_ or S and *n* = 4, 6, or 8 for the most representative of them.

The most popular and widely studied of these cyclic oligomers is the calix[4]arene, constituted by four phenolic units linked by methylene bridges at the 2,6-positions and exhibiting an upper and lower rim from either side of a central annulus (Figure 2).

**Figure 3:**
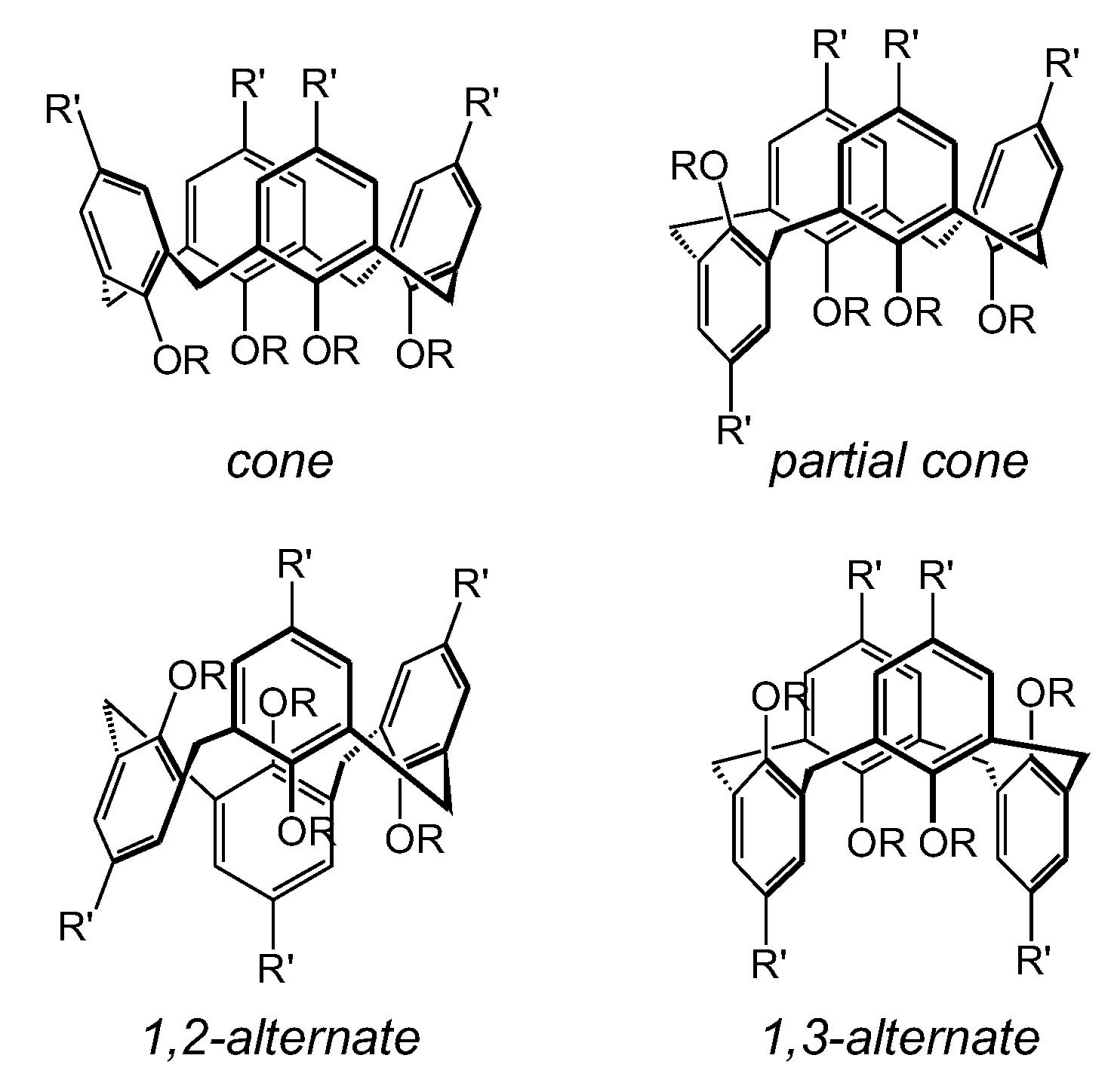
The four possible conformations of substituted calix[4]arene.

Moreover, this calixarenic skeleton displays the possibility to existing under four conformational isomeric structures: *cone*, *partial cone*, *1,2-alternate,* and *1,3-alternate* (Figure 3). Thus, the calixarene offers great possibility for structural modifications by the functionalization on the upper and/or lower rim with many kinds of functional groups. This versatility enables scientists to customize, to their taste, the properties of this molecular ring to provide the different chemical and physical properties required.

However, native calixarenes are not water-soluble; thus, exploration of their potential biological applications was quickly trained towards the development of water-soluble structures [56]. The first water-soluble calixarene appeared in 1984 with Arduini et al. and the introduction of four acetate groups on the lower rim of *para-tert*-butylcalix[4]arene [57]. However, the solubility of alkali and ammonium salts of the acid in water is only between 5 × 10^−4^ and 5 × 10^−3^ M, depending on the cation used (Na^+^ < K^+^ < Cs^+^ < NH_4_^+^ < Li^+^). Then, in 1988, Gutsche introduced the carboxylate functions on the upper rim [58]. In 1984, Shinkai et al. described the synthesis and application of sulfonated calixarenes, for example, [59,60,61], obtained by electrophilic substitution of previously detertiobutylated calixarene. The emergence of phosphonate derivatives in 1989 [62] was followed by ammonium via introduction of NH_2_ groups in the *para* position on a phenolic nucleus [63,64] or by aminodehydroxylation [65,66] of phenolic functions.

The addition of hydrophilic properties has also been envisaged by the introduction of sugar groups [67,68], polyhydroxy-amines [69], or PEG arms [70]. More recently, the development of water-soluble guanidinium calixarenes was introduced by Dudic et al. [71], and then by Mourer et al. [72].

## 5. Overview of Functionalization of Calix[4]arene

The different nature of the two rims available on the calixarene skeleton allows for selective functionalization on the upper rim (functionalization of the para carbon of the aromatic nucleus) or on the lower rim (functionalization of the phenolic hydroxyl) (Figure 2). Concerning the upper rim, it can be modified by aromatic nucleophilic substitution, via halogenation [73,74], nitration [75,76], sulfonation [59,61,77], acylation [78,79], or other means. In addition, on the native, *para*-tert-butylated calixarene, it is possible to completely or selectively detertiobutylate by first passing through the protection of the OH of the phenolic rings on which it is desired to retain a *para*-substitution [80]. On the other hand, the lower rim allows, via the reactive hydroxyl groups, the introduction of various arms by formation of ether or ester bonds [81].

In the case of calix[4]arene, the lower rim has four hydroxyl groups engaged in a network of intramolecular hydrogen bonds favoring the conical conformation (Figure 3). These strong hydrogen bonds lead to different pKa values for the hydroxyl groups involved in this ring [82]. This allows the establishment of various regio- and stereo-selective functionalization procedures on the lower crown. It has been shown that by implementing a carrier ion effect (“template effect”) relating to the nature and strength of the base used, it is possible to stereospecifically incorporate different substituents on the lower part of the calixarene. It is thus possible to substitute in the lower rim a calix[4]arene with 1 to 4 groups and, moreover, for bis-functionalization, a possibility of regioselectivity in the *1,2-adjacent* or *1,3-alternated* position [83,84,85,86,87,88,89,90,91,92].

## 6. Cytotoxicity of Calix[*n*]arenes

To develop biomedical applications, research into strong, possibly deleterious, interactions between the design molecular object and the biological target (cell, protein…) must examine the toxicity of various biological entities that these objects could potentially encounter before reaching their target. This is the case for the calixarene derivatives, more particularly the water-soluble ones, with the increase in interest which devoted to them for various biological applications and potential clinical studies. Relatively rare some twenty years ago, these studies are beginning to appear, and almost all converge towards the fact that this structure seems to have no toxicity or immunogenicity [93,94].

For example, the *para*-sulfonatocalix[*n*]arenes, widely used for numerous biomedical applications (anti-viral, anti-thrombotic, protein complexation, anti-coagulant…) exhibit an innocuous character with regard to studies conducted until this date [35].

**Figure 4:**
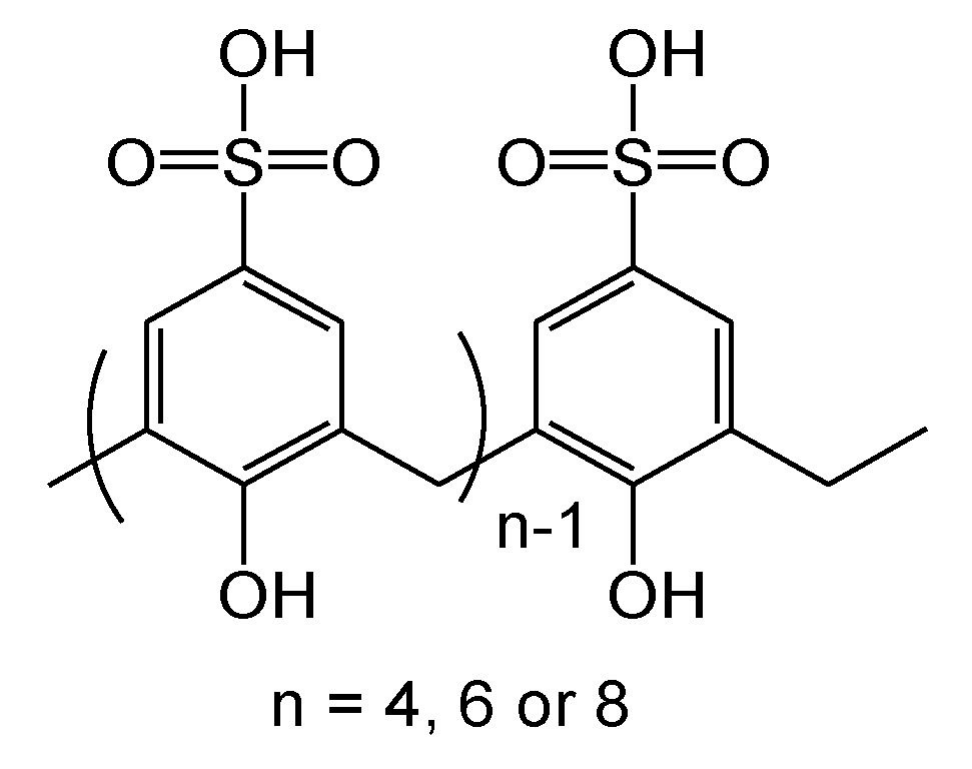
Structure of *para*-sulfonated calix[4, 6, or 8]arenes [95].

Therefore, for the first time, Da Silva et al. studied the toxicity of some sulfonated calixarenes (Figure 4) on erythrocytes for their haemolytic properties [95]. These, functionalized at the lower rim by the 2-carboxy methoxy group, 2-amido methoxy group, or 2-amino ethoxy group, and designed for some complexation properties of bovine serum albumin (circulating physiological protein) [96], present a maximum of 30% of haemolysis (evaluated by release of haemoglobin from lysed cells) for the *para*-sulfonatocalix[8]arene at the high concentration of 200 mM (about 300 g/L of blood). The corresponding calix[4 and 6]arenes are much less toxic, with 0.5 and 8.0% of haemolysis, respectively. These results show a very low toxicity on red blood cells of sulfonated calixarenes.

The same *para*-sulfonatocalix[*n*]arenic structures were tested for the viability and stimulation of neutrophils involved in immune response. Cell viability was not affected and the calix[*n*]arenes studied did not induce activation of NADPH oxidase in neutrophils, and did not reduce their responses to an external stimulus [97].

The first in vivo study of the distribution and pharmacokinetics of 35S-labelling *para*-sulfonatocalix[4]arene was conducted on mice by Coleman et al. in 2008 [98]. The experiment showed no acute toxicity for single injected doses (equivalent to 2–5 g in humans), no crossing of the blood–brain barrier, nor passing into muscles, and the molecule is quickly eliminated in mouse urine. It is unlikely that the 35S-*para*-sulfonatocalix[4]arene is metabolized, considering the lack of accumulation in the liver.

A study for using it as an anticancer drug delivery vehicle showed that this same structure presents no cytotoxicity against the human ovarian carcinoma cell line A2780 and its equivalent cisplatin-resistant cell line A2780cis [99].

In 2010, Geller et al. proposed a study on the antiseptic properties of *para*-sulfonatocalix[4]arene and its bis-bithiazolyl derivative against human coronavirus 229 E [100]. These two calixarenes did not show any cytotoxicity on the L-132 epithelial cell line, contrary to chlorhexidine and hexamidine, used as antiseptic references.

The toxicity of a fluorescent probe derived from an aminocalix[4]arene was studied on Chinese hamster ovary cells (CHO) and HL50 cells (i.e., human leukemia cells) [101]. The fluorescent water-soluble calix[4]arene and its unlabeled parent did not show more toxicity than phosphate-buffered saline (PBS) over a range of concentrations with IC_50_ of 82 and 81 mM, respectively.

A wide series of anionic calixarenes—*para*-methylcarboxylate, *para*-sulphonate, and *para*-methylphosphonate and their bis-bithiazolyl derivatives—were evaluated as anti-HIV compounds by Mourer et al. [102]. All of the nine water-soluble calixarene derivatives evaluated on the two cell lines MT4 (HTLV-I-transformed T-cell line) and CEM-SS (T-cell line expressing the CXCR4 co-receptor) display very weak or no toxicity, as the CC_50_ was not reached at 100 µM. A similarly very low toxicity level was also detected on PBMC (T-cell line expressing the CCR5 co-receptor) for the majority of substances tested.

Since 2006, Mourer et al. have examined systematically the cellular toxicity of calixarenes designed for the research on biological applications. They showed that most calixarene structures are often devoid of in vitro cellular toxicity, particularly on HaCaT cells (spontaneous transformed aneuploid immortal keratinocyte cell line) and/or on MRC-5 cells (fibroblast-like human embryonic lung cells) [72,103].

## 7. Antibacterial Activities of Calix[*n*]arenes

The calixarenic structure is endowed with great versatility, and this is why it is used in a wide variety of fields. The most interesting features are the multiple functionalizable positions and the access to multivalence. Multivalency is, for example, a key element in generating the strongest and most specific interactions possible [104,105,106].

This last is widely used for new drug research, for example: simple use as a carrier of multiple active pharmaceutical ingredients (API) (identical or different) in the case of development of prodrugs; the genesis of an environment conducive to the complexation of metals in the search for compounds capable of disrupting the activity of metalloenzymes or the formation of toxic metal complexes; and research on therapeutic activities related to strong interactions with proteins, enzymes, or nucleic acids [107].

As the versatility of calixarenes is very wide, it is possible to optimize the design of a multivalent ligand to improve the desired activity. For example, the length of the bond between the calixarene and the group of interest will often be a very important and critical point: a calix[4]arene could lead to spatial constraints and greater interactions than a calix[8]arene; however, this last, more fluxional, could allow more adaptability face to a given target. Thus, using this central skeleton, researchers can very precisely tune the nature, number, distance, or orientation in space of any type of functionality in such a way as to best match a biological target as finely as possible.

### 7.1. Intrinsically Active Calixarenes

Since the 1950s, researchers have sought to functionalize calixarene crowns with various types of functional groups, sometimes known to already possess a biological activity. The objective is often to take advantage of the multivalence provided by the macrocycle and to envisage a synergy of action. It appears very clearly in certain studies, by comparison with the constitutive monomers of calixarene, that the latter allows a marked improvement in the activities sought, fully justifying its use.

**Figure 5:**
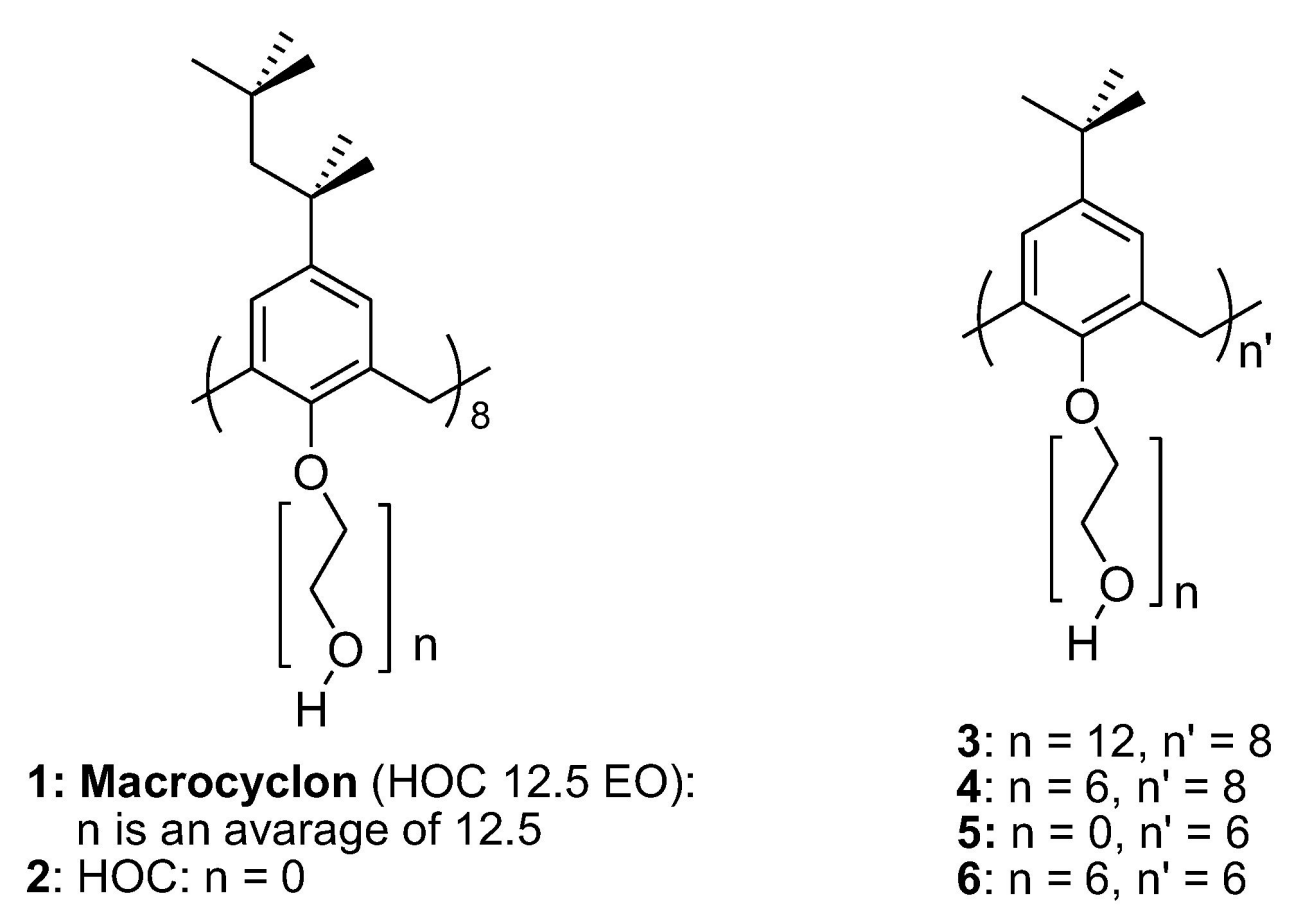
Schematic structures of the new antimycobacterial calixarenes [111,112].

The first direct antibacterial activity of calixarene was reported in 1955 by Cornforth et al. [108,109]. An important study was carried out on macrocyclon, alternatively named HOC 12.5 EO (**1** Figure 5), constituted from macrocyclic phenol (calix[8]arene), which for brevity is termed HOC, and which was prepared from tert-octylphenol and formaldehyde by a modified Zinke–Ziegler procedure, and by reacting under basic conditions with ethylene oxide (EO). The four resulting polyoxyethylene ether chains have an average chain length of 11–12.5 units (HOC 12.5 EO) [109,110].

The macrocyclon enters macrophages via endocytosis and disrupts their lipid metabolism by inhibiting triglyceride lipase and phospholipase, blocking bacterial growth. While the growth of *Mycobacterium tuberculosis* (H_37_Rv strain) in infected macrophages is inhibited by macrocyclon (bacteriostatic), this is stimulated by HOC-60 (derived from the macrocyclon with a much longer polyoxyethylene chain) [111,112].

The study of compounds **2** to **6** has shown that a polyethylene glycol (PEG) chain with 6 units (**4** and **6** Figure 5) is sufficient to provide a high antimycobacterial activity and that the change to 12 units (**3** Figure 5) does not bring significant improvement. Likewise, hexamer **6** and octamer **4** exhibit similar activities, suggesting that the size of the calixarenic crown is not critical. However, in the case of non-functionalized compounds **2** and **5**, the size of the cavity seems to intervene, **2** having a slight antimycobacterial activity while **5** is inactive. The authors showed additionally that macrocyclon significantly affected mycobacterial growth in murine macrophages by a mechanism involving L-arginine metabolism and iNOS activity [113].

**Figure 6:**
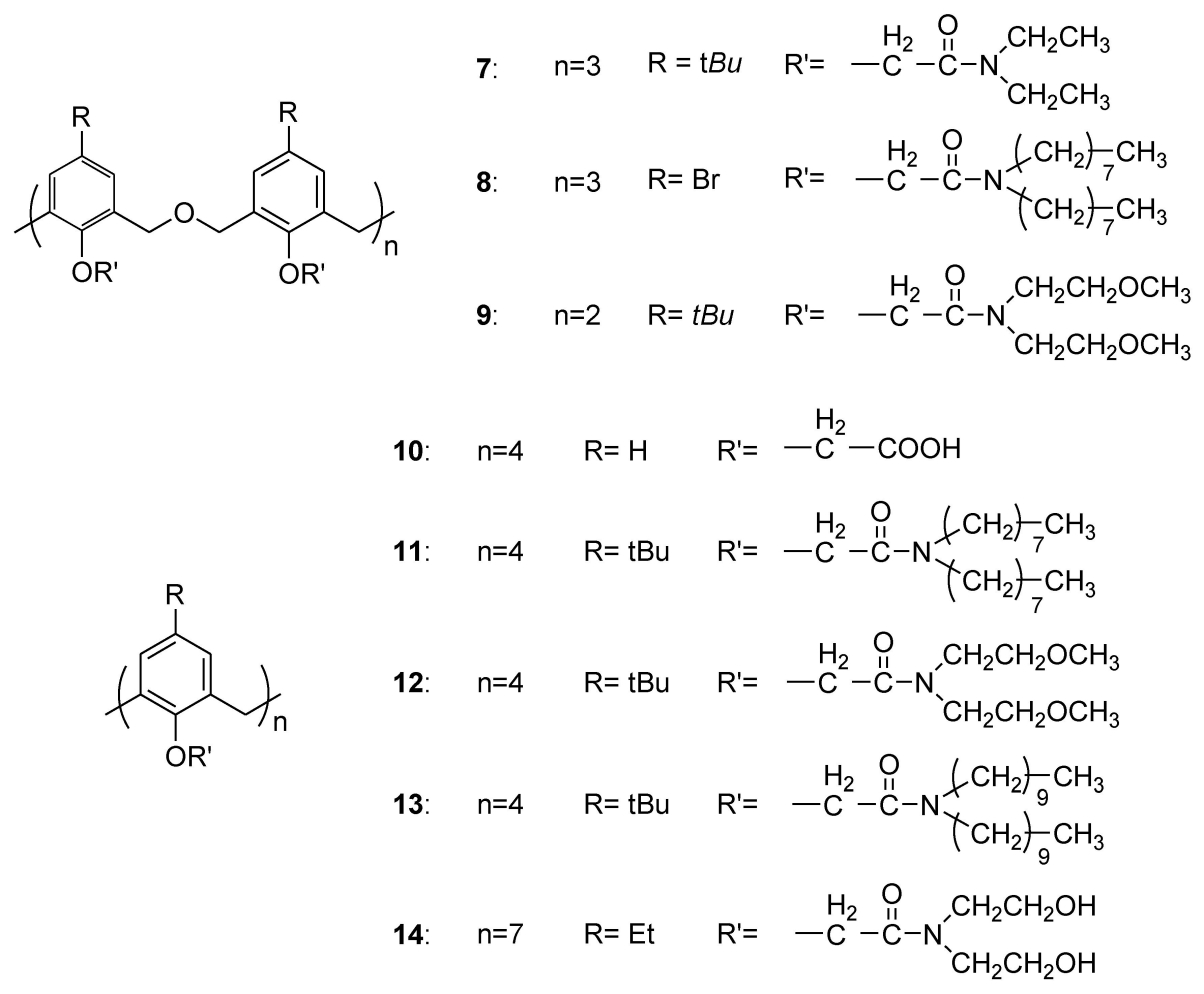
Structure of some calixarenes and oxacalixarenes presenting antibacterial activities [114].

In 1995, S. J. Harris patented a large screening of more than 200 molecules (calixarenes, oxacalixarenes, cyclotriveratrylene derivatives…) for their anti-HIV, anti-cancer, antibacterial, and antifungal activities [114]. Only some antibacterial activities were reported for a series of calixarenes and oxacalixarenes having amide functional groups in the lower rim (Figure 6).

The bacteria chosen are those usually employed in routine screening for potential antibacterial activity in the 1990s: *Escherichia coli*, *Pseudomonas aeruginosa*, *Klebsiella pneumoniae* (lactamase producing strain), *Bacillus subtilis,* and *Staphylococcus aureus* (Table 1).

The structures tested do not show good activities, i.e., from 1 to 10 mg/mL, corresponding to MICs 50 to 100 times greater than that obtained for the different references used in the study (ampicillin, tetracycline, or erythromycin MIC values are ≤0.1 mg/mL). There does not seem to be any selectivity towards the structure of the bacteria (Gram-positive or -negative). Compounds **7**, **11**, **12,** and **13** display, for example, an activity on both Gram types. Similarly, the results do not allow clear determination of a structure–activity relationship. Whether it is a calixarene crown (4 or 7 phenolic units) or oxacalixarene, the activities seem more or less random. Functional groups *N*,*N*-bis-alkyl- (**7**, **8**, **11**, **13**), *N*,*N*-bis(2-hydroxyethyl)- (**14**), or *N*,*N*-bis(2-methoxyethyl)-ethanamide (**9**, **12**) in the lower rim do not appear to lead to any particular activities.

Casnati and coworkers, in 1996, used the calixarenic platform bridged in the upper rim by D- or L-alanine units and the diethylentriamine segment as vancomycin antibiotic mimics [115]. Isolated in 1950, the glycopeptide vancomycin was clinically used in 1959 against *S. aureus* both sensitive and resistant to penicillin or methicillin. Some studies determined its mode of action via interaction between his peptide moieties and the cell wall mucopeptide precursors terminating in the sequence –D-alanyl-D-alanine. This hydrophobic interaction inhibits the growing of the cell wall and thus the bacterial wall.

**Scheme 1:**
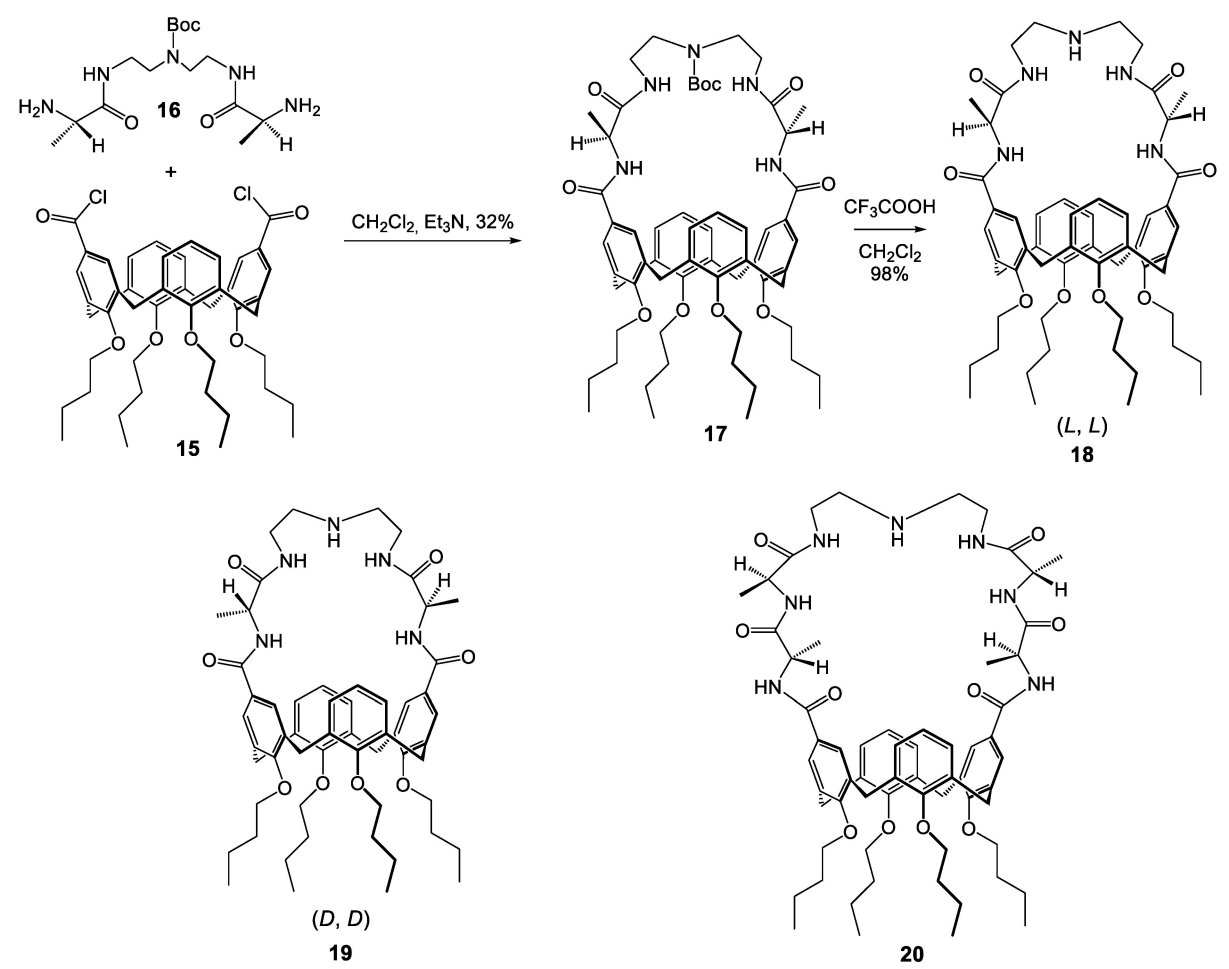
Synthetic way leading to the peptidocalix[4]arene **18** and structures of its analogues **19** and **20** [115].

An example of the synthesis of the short-bridged (L, L) compound **6** is presented in Scheme 1. Condensation of calix[4]arene diacylchloride **1** and precursor **4** in high-dilution conditions gave the *N*-Boc protected macrobicyclic compound **5**, which was deprotected in acidic conditions to obtain the expected peptido-calixarene **6**. A similar procedure was used for the development of compounds **7** and **9** (Scheme 1).

These four new calixarenic structures (Scheme 1), and vancomycin taken as reference, were tested against various Gram-positive strains such as *S. aureus* (strains 663 (penicillin-sensitive), 853 (penicillin-resistant), and 1131 (methicillin-resistant)), *Staphylococcus epidermidis*, and *Bacillus cereus*. Although slightly inferior to vancomycin (2 µg/mL), the calixarenes studied, notably compounds **18** et **19**, show good activity against the MRSA (methicillin-resistant *Staphylococcus aureus*) strains (4 to 8 µg/mL) (Table 2).

The two enantiomers **18** and **19** present the same range of activities. Moreover, the Boc-protected compound **17** and the calixarene with a largest peptide bridge **20** display a reduction in antibacterial activity. In parallel, the peptide precursors used for obtaining calixarenes **18**, **19,** and **20** are inactive, confirming the importance of the calixarenic skeleton to maximize the interactions with the sequence –D-alanyl-D-alanine. These results were confirmed by binding studies with the classical model *N*-acetyl-D-alanyl-D-alanine for cell wall peptidoglycan termini via NMR analysis [115,116,117].

**Figure 7:**
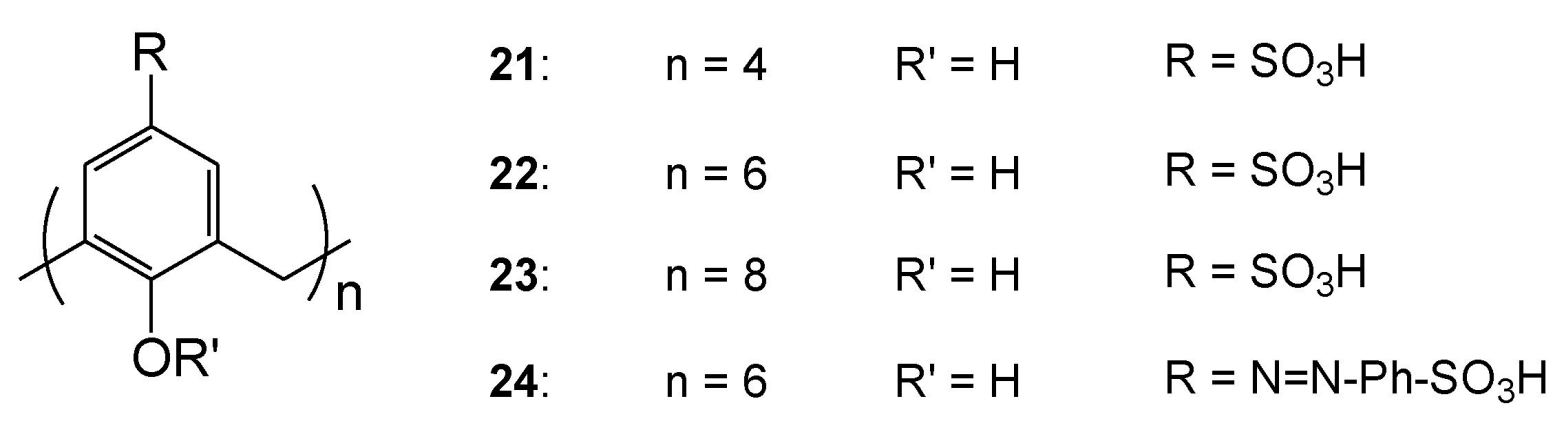
Calixarenic structures **21** to **24** [77,109].

In 2002, Lamartine et al. published a study of 57 calixarenes having different side chains, moieties, and/or substitution groups against a diverse set of plant pathogen bacteria and fungi [118]. Firstly, antibacterial activity was performed against the specific Gram-positive genus *Corynebacterium* to select the calixarenic active structures according to the inhibition diameter. From this first screening, only seven structures exhibited antibacterial activity. Then a second antibacterial test was performed to extend the activity as a function of calixarene concentration (0.001 to 1%, i.e., 10 to 10,000 ppm (part per million)). Finally, only sulfonated (basic form) compounds **21** to **24** (Figure 7) exhibited slight antibacterial activities. These sulfonate-calixarenes **21**, **22,** and **23** were easily obtained by *ipso*-sulfonation [77] and by diazo-coupling for the **24** derivative [119]. Unfortunately, these structures are active above 1% concentration (**21**, **22**, **23**) and at best at 0.1% (1000 ppm) for the derivative **24**. These activities seem not effective enough for pesticide development (considered effective at less than 100 ppm).

**Scheme 2:**
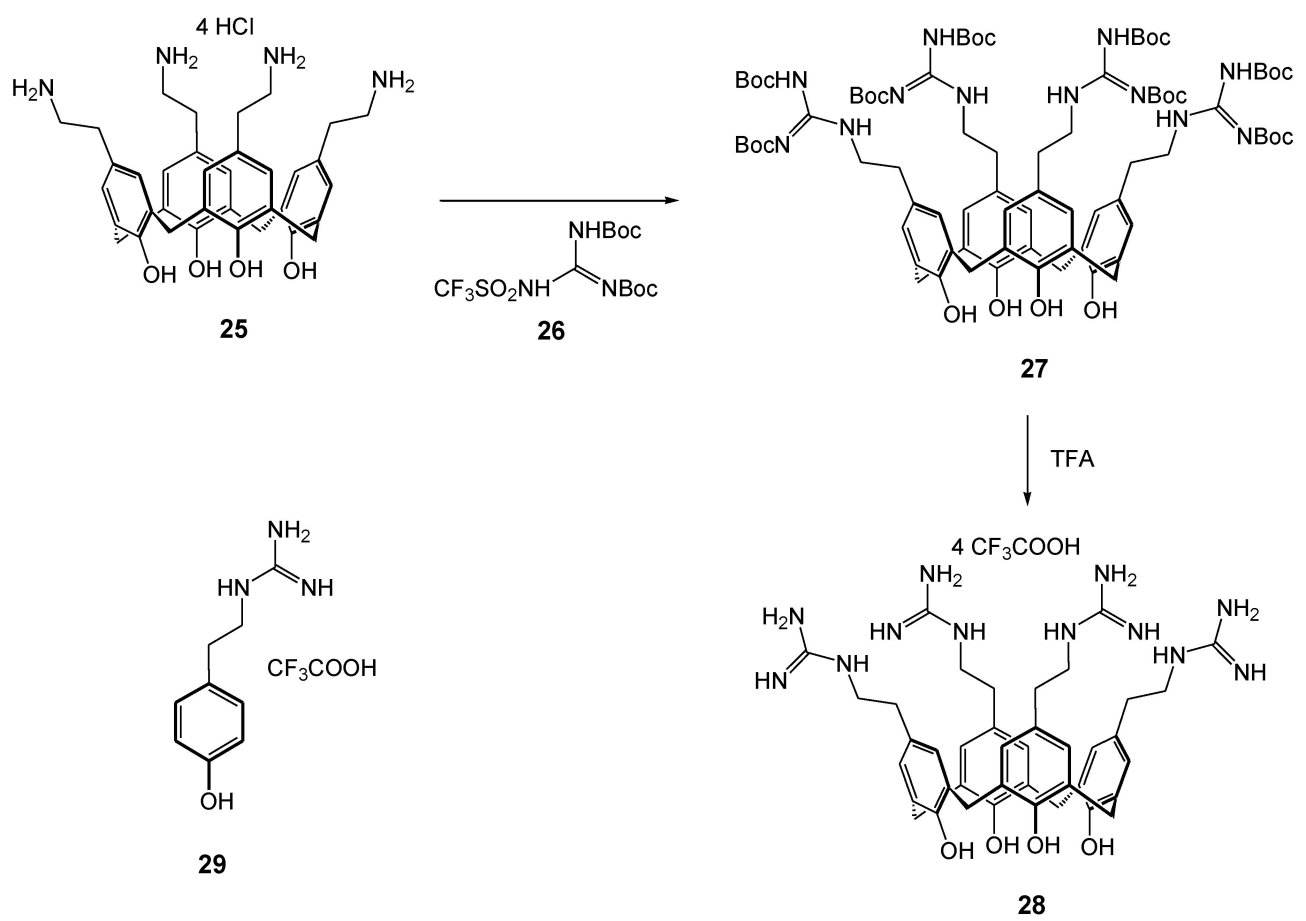
Synthetic pathway to compound **28**, and representation of its monomer analogue **29 [72]**. Boc: tert-Butoxycarbonyl.

In 2006, Mourer and co-authors began a large series of studies on the antibacterial activities of calixarenes variously substituted in the upper and/or lower rim. The object of the first study was the *tetra*-*para*-guanidino-ethylcalix[4]arene **28** (Scheme 2), which exhibits an organization of positive guanidinium charges on the upper rim and displays a significant antibacterial activity (MIC <8 µg/mL) against the bacterial reference strains *E. coli* (ATCC 25922), *S. aureus* (ATCC 25923 and ATCC 29213), *Enterococcus faecalis* (ATCC 29212), and *P. aeruginosa* (ATCC 27853) (Table 3) [72], but also on the clinical isolates penicillinase-producing *E. coli* (EcR1), methicillin-resistant *S. aureus* (MRSA), vancomycin-resistant *E. faecium* (EfR1), vancomycin- and teicoplanin-resistant *E. faecalis* (EfR2), and *P. aeruginosa* overexpressing efflux pumps (PaR1) (Table 3). [120]. The initial hypothesis was based on the fact that most bacteria are negatively charged, and that introduction of positive charges in a constrained manner using the organization capacity of calixarene could lead, via spatially organized electrostatic interactions, to a modification of the ionic surface properties, inducing a disorganization of it and thus to a deleterious effect for bacterial cells. In this study, the authors propose to compare the activity of the calixarene structure with its constitutive monomer **29** (Scheme 2) on the assumption that the high organization of calixarene derivatives should lead, with regard to the monomeric analogue, to an efficient synergistic effect in ionic interaction with the bacterial surface, resulting in an antibacterial behavior.

Calixarene **28** was obtained according to Scheme 2, with *tetra*-*para*-ethylaminocalix[4]arene **25**, which was synthesized by a modified Gutsche procedure [64]. Addition of di-Boc-triflylguanidine **26** allows the *octa*-Boc species **27** formation, which was finally treated with trifluoroacetic acid to give the *tetra*-guanidinium salt **28** with overall yield of 52%.

The authors thus show the importance of the calixarenic skeleton: its ability to organize in space and to constrain cationic functions. Switching from the monomer **29** (inactive, MIC > 512 µg/mL) to cyclotetramer results in a very strong gain of activity on all the strains (4 to 32 µg/mL on *E. coli* and *E. faecalis* respectively). The very good activity of **28** on Gram-positive and Gram-negative bacteria, suggests a broad antibacterial spectrum.

Additional tests showed that **28** exhibits a rapid concentration-dependent bactericide activity (in 2 to 4 h of contact). A minimal bactericidal concentration against *E. coli* of 8 × MIC, and 4 × MIC on *P. aeruginosa* and *S. aureus*, was observed [121]. *Tetra*-*para*-guanidino-ethylcalix[4]arene **28** presented an antibacterial spectrum similar to hexamidine HX or chlorhexidine CHX, well-known antibacterial agents used as references [120]. Both *tetra*-*para*-guanidino-ethylcalix[4]arene **28** and its constitutive monomer **29** showed no apparent cytotoxic effect against eukaryotic cells (human keratinocytes (HaCaT) and human pulmonary embryonic fibroblasts (MRC-5)) (>256 µg/mL) even up to 168 h of cell exposure, contrary to hexamidine, with loss of cell viability seen after 24 h of cell exposure (IC_50_ = 36–37 µg/mL) for both HaCaT and MRC-5 cell lines [72].

A complementary study on this cationic calix[4]arene **28** was carried out on a large series of multiresistant bacteria from clinical isolates: 39 MDR Gram-positive bacteria (15 *S. aureus*, methicillin-resistant (MRSA) or methicillin-sensitive (MSSA), 12 coagulase-negative *staphylococci* (CoNS), resistant or susceptible to methicillin, 14 *Enterococcus* spp., with or without *van* genes) and 30 MDR Gram-negative bacteria (20 *Enterobacteriaceae*, with or without Extended Spectrum Beta Lactamase (ESBL) and 10 non-fermenting bacilli). The results showed a maintained activity on almost all of the strains and the authors advance a probable action of **28** at the level of the bacterial cell wall [122].

Faced with the excellent activity of this cationic derivative and the fact that it is not stopped by the resistance mechanisms commonly used by bacteria, the authors tried to understand the mechanism of action with a first study via microelectrophoresis [123] in order to obtain a global evaluation of the interfacial properties of bacteria with or without drug treatment. These experiments carried out on *P. aeruginosa* after 24 h of exposure to the drug showed a significant decrease in electrophoretic mobility, reflecting a strong increase in the permeability of the walls and/or a specific adsorption of calixarenes on the surface justifying a wall effect. In order to confirm this impact on the cell wall, they carried out a study via Atomic Force Microscopy (AFM) in order to visualize any alterations in the bacterial cell wall. The first images obtained on *P. aeruginosa* effectively show alterations in the wall after 24 h of contact with calixarene as well as very significant cell fragility upon simple contact with the AFM tip compared with untreated cells [123]. The same study showed that the constitutive monomer **29** does not cause any alterations.

In a second step on *P. aeruginosa* ATCC 27853 and PaR3 (i.e., a MDR strain), a comparative study was carried out between ticarcillin, tobramycin (to which PaR3 is resistant), and calixarene **28** [124]. If the general appearance of sensitive bacteria is well modified in the presence of ticarcillin and tobramycin, the resistant strain is not impacted. On the other hand, treated by **28**, the two strains present alterations of the wall, appearance of perforations, and surface inequalities (from 0.2 ± to 0.04 nm for untreated bacteria to 1.0 ± 0.2 nm for cells treated with **28**).

The results also showed that on the resistant strain of *P. aeruginosa* (i.e., PaR3), **28** had a radical effect on the elasticity of the wall not encountered with the reference antibiotics. For example, the PaR3 strain saw its modulus of elasticity drop from 520 ± 100 kPa to 300 ± 66 and 252 ± 61 kPa after treatment with ticarcillin and tobramycin, respectively, but fell to 76 ± 28 kPa during treatment by **28**.

Other experiments conducted on phospholipid bilayers showed that **28** created perforations in the latter (0.5 to 1 mm in diameter), agreeing with the observations made on the cells treated with **28**.

In 2014, Grare and Duval patents the use of calixarene *tetra*-guanidinium salt **28** in the treatment of bacterial infections in combination with some known antibiotics [125]. Considered as possible way to bypass the antibiotic resistances, the bi-therapy could ideally generate a synergistic effect between two drugs. The objective of the study is to determine, in vitro, the possible benefit of the association of **28** with various antibiotics conventionally used in practice, with different targets of action. They performed an important screening against sensitive *E. coli* ATCC 25922, *S. aureus* ATCC 29213, and *P. aeruginosa* ATCC 27853 reference strains, three clinical isolates by species presenting all various resistance profiles to antibiotics conventionally used in practice. Calixarene **28** was evaluated in association with 12, 11, and 10 antibiotics against *E. coli*, *S. aureus* and *P. aeruginosa,* respectively. Initially, the results allowed the absence of antagonism on all the associations evaluated and the activity of some antibiotics seems restored for all clinical isolates tested. An important impact on *P. aeruginosa* clearly appears, as it exhibits a greater sensitivity than *E. coli* or *S. aureus* to the association of drugs. Indeed, it is the strain for which the most synergistic associations have been recorded, even against clinical isolates displaying carbapenem resistance, strains classified as a priority pathogen by the WHO in the search of new antibiotics [126]. The derivative **28** makes it possible to confer de novo a certain level of susceptibility to antibiotic on bacterial strains having a natural or acquired resistance to said antibiotic.

More recently, Korchowiec et al. have studied the behavior of calix[4]arene guanidinium salt **28** and its constitutive monomer **29** against models of lipid membranes (monolayers) by simulation of molecular dynamics and measurement of surface pressure. The studies were carried out on a zwitterionic monolayer (1,2-dimyritoyl-sn-glycero-3-phosphocholine: DMPC) as a eukaryotic cell membrane model and negatively charged (1,2-dimyritoyl-sn-glycero-3-phospho -L-serine: DMPS) as a bacterial membrane model [127]. Although no effect has been demonstrated on the (zwitterionic) eukaryotic cell membrane model, the (anionic) bacterial cell membrane model is significantly affected in the presence of **28**.

Beyond the fact that the charge–charge interactions play an important role in the adsorption on the negatively charged phospholipid heads, leading to an accumulation of molecules at this level, it would seem that **28** also penetrates into the hydrophobic part of the monolayer, in particular via its hydrophobic lower rim. A reversal of the orientation of *tetra*-guanidinium upon penetration into the lipid layer, minimizing harmful interactions, could also play a role in the demonstrated antibacterial activity. Under the same study conditions, the monomer **29** appears to be rapidly removed from the monolayer.

**Scheme 3:**
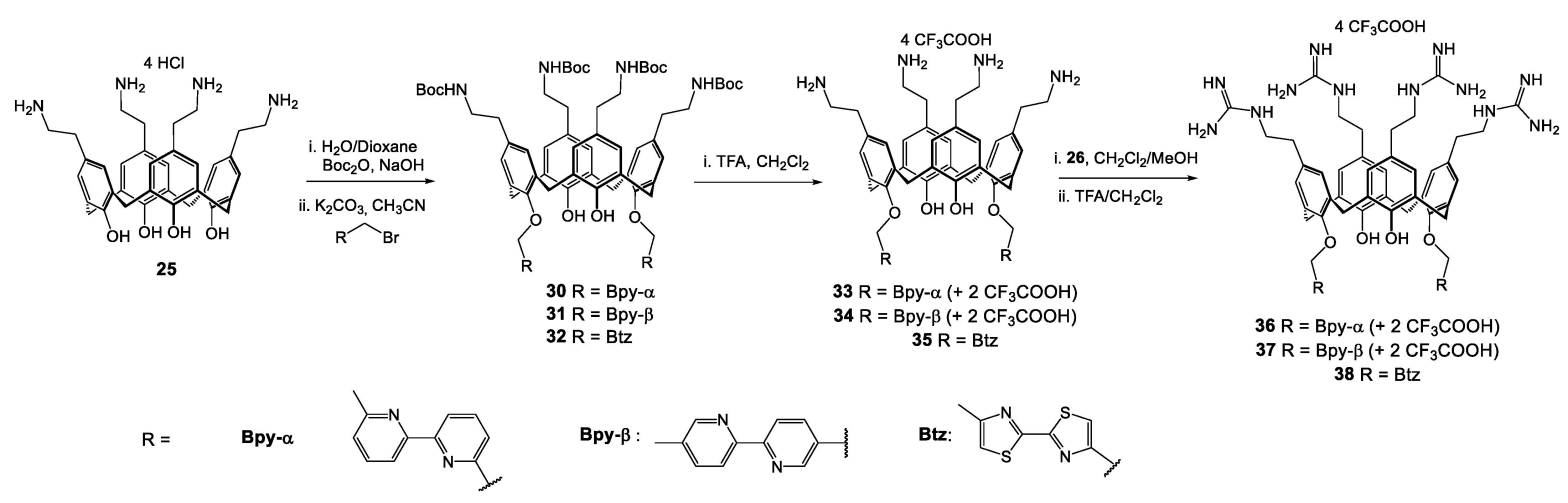
Synthetic pathway to *tetra*-guanidinium bis-heterocyclic compounds **36** to **38** [128]. Boc: tert-Butoxycarbonyl, Bpy-α: 6-[6′methyl-2,2′-bipyridine], Bpy-β: 5-[5′-methyl-2,2′-bipyridine], Btz: 4-[4′-methyl-2,2′-bithiazole], TFA: trifluoroacetic acid.

In order to increase the antibacterial activities of tetra-guanidino calix[4]arene **28**, Mourer et al. developed, in 2009, some derivative carriers of bis-heterocyclic arms in the 1,3-alternating position of the lower rim [128]. In addition to the electrostatic interactions between the positive guanidinium charges and the negatively charged wall of the bacteria, they hope, by introducing an amphiphilic character to their structure, to generate a disorganizing insertion in the bacterial wall. In addition, the complexing capacity of bis-heterocycles could allow the capture of metal ions necessary for bacterial multiplication (metalloenzymes) or the genesis of toxic metal complexes for the bacterial cell. These structures were obtained according to Scheme 3. The *tetra*-salt of ammonium **25**, once protected under carbamate form, undergoes the introduction of bis-heterocyclic units in the *1,3-alternate* positions in acetonitrile and K_2_CO_3_ as base (compounds **30**, **31**, **32;** Scheme 3). The bis-heterocyclic compounds obtained were deprotected (**33**, **34**, **35;** Scheme 3) and the introduction of guanidine moieties was performed in the same way as described above using the di-Boc-triflylguanidine **26**, followed by a final elimination of the Boc protective groups in TFA conditions to give the expected *tetra*-guanidinium salts **36**, **37**, and **38**.

The microbiological study consisted of the determination of active concentrations on Gram-positive and Gram-negative reference strains as well as the in vitro cellular toxicity on eukaryotic cells of the MRC-5 type (Table 4). Unfortunately, even if the activities of bis-heterocyclic derivatives are preserved with MICs of 10 to 20 µg/mL close to the reference derivative **28**, the introduction of bipyridyl or bithiazolyl arms does not appear to bring about a significant modification of the antibacterial behavior. In addition, a much greater toxicity appears on the eukaryotic cell, in particular for the derivatives **36** and **38** with IC_50_s of 16 and 64 µg/mL, close to the antibacterial values, leading to weak selectivity indexes.

In the same way as with the introduction of positive charges, negative charges also provide hydrophilicity to the calixarenic platform. Thus, the same team published in 2012 the synthesis and antibacterial evaluation of water-soluble derivatives incorporating carboxylate groups in the upper rim and bis-heterocycles in the *1,3-alternate* positions on the lower rim of calix[4]arene [129].

**Scheme 4:**
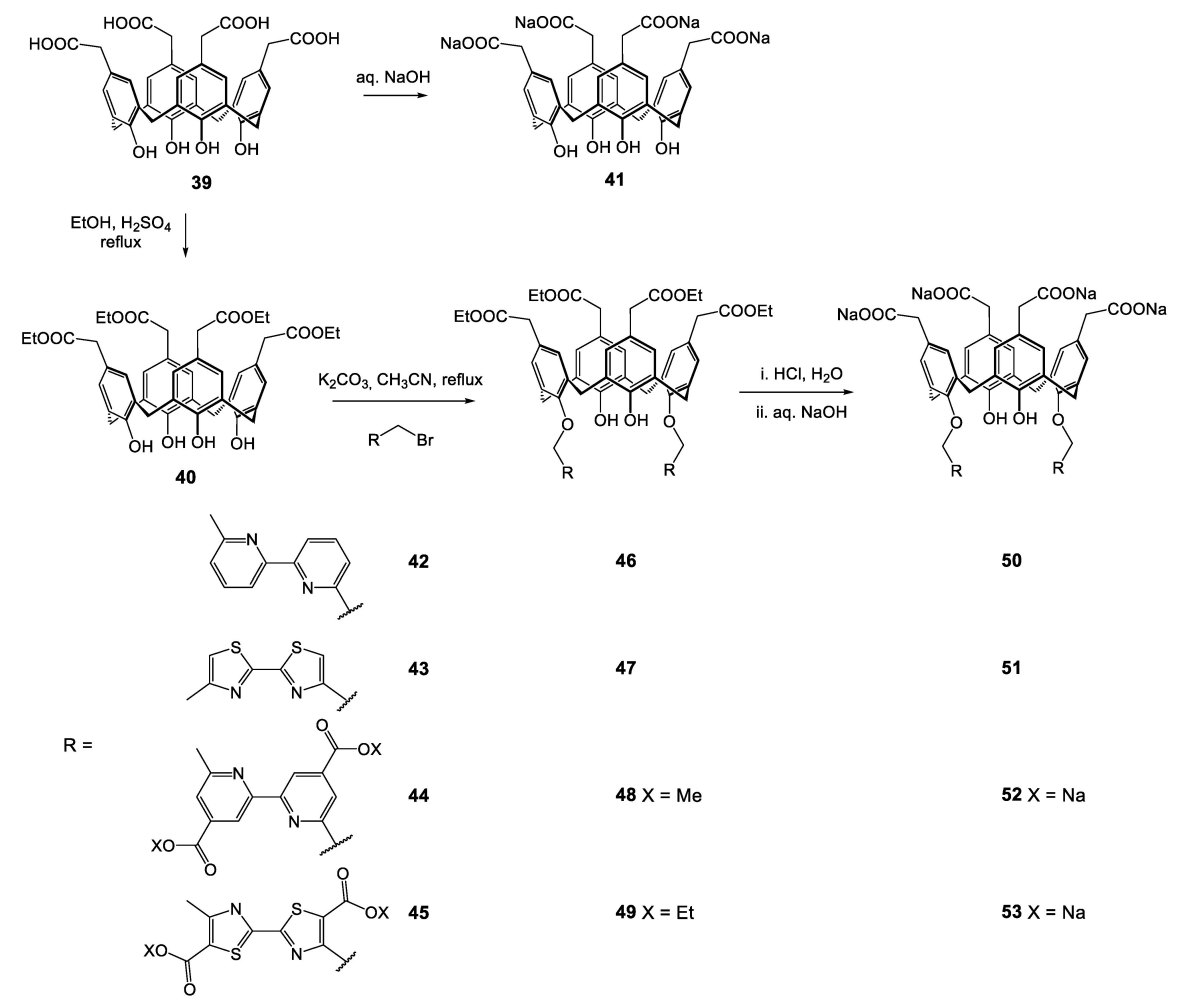
Synthetic pathway to water-soluble calix[4]arenes incorporating carboxylate groups **50** to **53** [129].

The tetra acetic acid derivative **39** and its tetraethylester **40** were prepared according to the procedures of Gutsche and co-workers [64,130]. Introduction of the heterocyclic subunits in alternate positions on the lower rim of **40** was carried out by the method of Mourer et al. [102] by reaction of bromomethyl derivatives **42** to **45** in MeCN and K_2_CO_3_ as base (Scheme 4). The sodium salts were obtained under a previously described procedure [131], namely that the tetra-esters **46**, **47** and octa-esters **48**, **49** were saponified with NaOH in hydro-alcoholic medium and precipitated under acidic form with aqueous HCl. Then, a titration with NaOH gave the expected sodium salt derivatives **50** to **53**.

Conducting against Gram-negative (*E. coli* ATCC 25922 and *P. aeruginosa* ATCC 27853) and Gram-positive (*S. aureus* ATCC 25923 and ATCC 29213, and *E. faecalis* ATCC 29212) reference strains, the MIC determination showed that these carboxylate water-soluble calixarenes display no antibacterial activity under 256 µg/mL (128 µg/mL for **53** on *S. aureus* ATCC 25923).

The authors report that they observe, at this stage of their studies, an effective discrimination between the antiviral [102] and antibacterial activities of these compounds, directing the negatively charged derivatives towards antiviral activities and the positively charged derivatives towards antibacterial activities.

**Figure 8:**
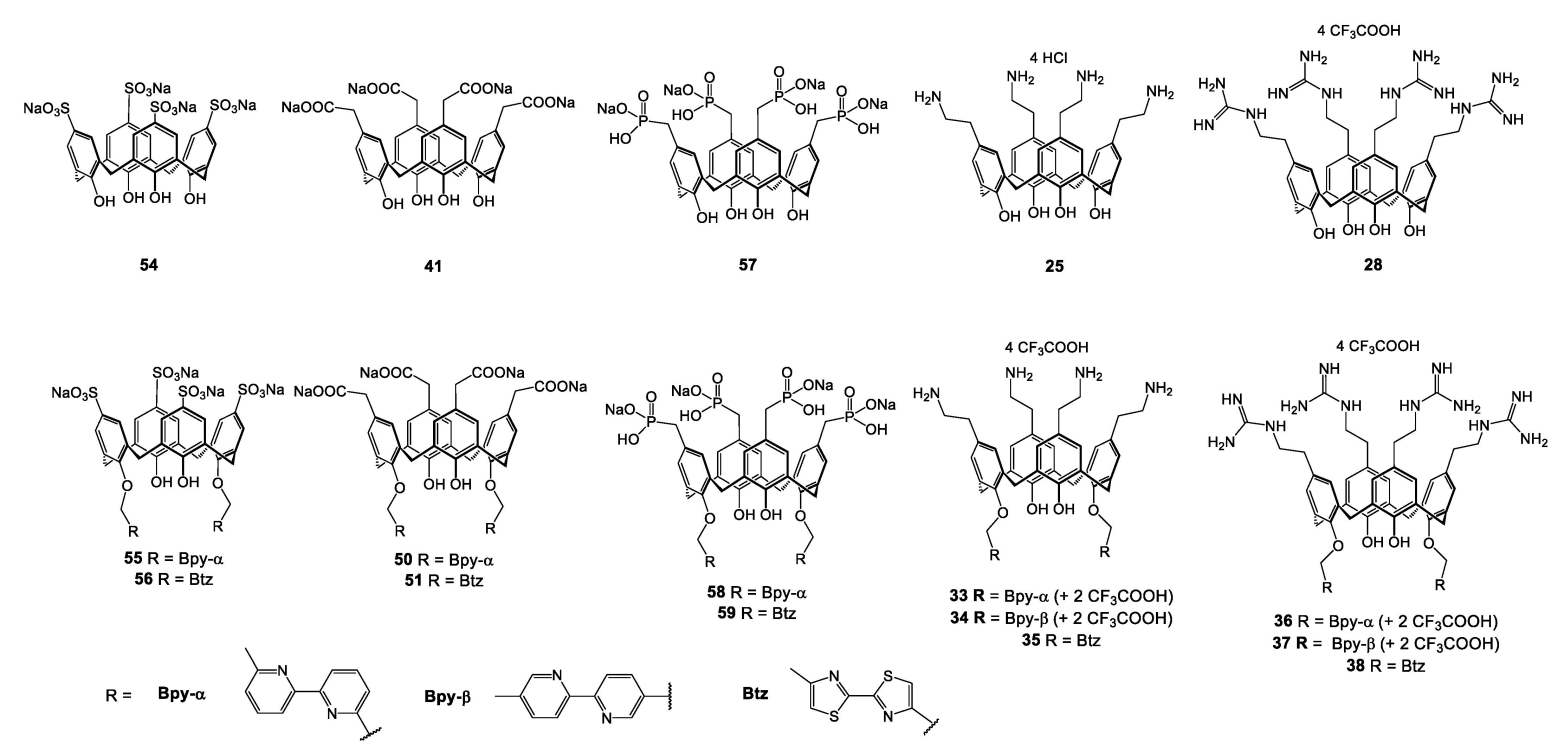
Negatively and positively charged calix[4]arenes tested on *M. tuberculosis* [132]. Boc: tert-Butoxycarbonyl, Bpy-α: 6-[6′methyl-2,2′-bipyridine], Bpy-β: 5-[5′-methyl-2,2′-bipyridine], Btz: 4-[4′-methyl-2,2′-bithiazole].

On the strength of their results, Mourer and co-authors started a study of some of their calixarene derivatives (Figure 8) on a particular strain: *Mycobacterium tuberculosis*, with the resurgence of tuberculosis in sight, placing this bacillus at the foreground of the infections to fight [132].

A first screening at 10 and 1 μM was carried out against the isonicotinic acid hydrazide (INH)-sensitive *M. tuberculosis* H_37_Rv strain, with some of the anionic (sulfonates **54**, **55,** and **55**; carboxylates **41**, **50,** and **51**, phosphonates **57**, **58,** and **59**), and all cationic derivatives (ammoniums **25**, **33**, **34,** and **35**; guanidiniums **28**, **36**, **37**, and **38**). The authors confirmed their previous results [129], namely that anionic compounds were inactive. On the other hand, the results are more encouraging with cationic species, but variable, depending on the type of structure. In the amino family, the compounds α-bipyridyl **33** and bithiazolyl **35** are non-active to weakly active with 0 to 19% inhibition at 10 µM, respectively. The unsubstituted ammonium **25** and β-bipyridyl **34** derivatives displayed a better inhibitory property, evolving respectively from 76% and 81% at 10 µM to 27% and 63% at 1 µM. A similar behavior was observed for the guanidinium family. Exhibiting an inhibition of 10% at 10 µM, the α-bipyridyl **36** derivative is non-active. The unsubstituted guanidinium derivative **28**, the bis-(bithiazolyl) **38** and the bis-(β-bipyridyl) **37** display a high activity with respectively 97%, 90%, and 98% inhibitory concentration at 10 µM, but also at 1 µM concentration with 73%, 54%, and 62% inhibition.

After this first selection, the authors determined more precisely the active concentration via MIC determination against INH-sensitive (H_37_Rv) and -resistant (MYC5165) *M. tuberculosis* strains (Table 5).

Ammonium derivatives, unsubstituted and bipyridylated, display interesting activities with MIC values of 2.58 and 1.34 µg/mL, respectively, for **25** and **34** on H_37_Rv. Introduction of the guanidinium groups decreases the MICs with values of 1.0, 1.51, and 2.69 µg/mL, respectively, for **28**, **37,** and **38** on an INH-sensitive strain. In comparison, commercial antimycobacterial compounds INH, ethambutol, and streptomycin show MICs of 0.08, 2.0, and 0.4 µg/mL respectively.

In parallel against INH-resistant strain MYC5165, the same compounds exhibit very low MIC values, mostly sub-micromolar, while INH is active at 1.7 µg/mL. Indeed, the unsubstituted guanidinium **28**, β-bipyridyl **37**, and bithiazolyl **38** guanidinium compounds are responsible for remarkable MICs of 1.0, 0.75, and 0.17 µg/mL respectively.

The introduction of the guanidine groups at the upper rim in derivatives **28**, **37**, and **38** resulted, particularly for **38**, in more interesting activities compared with ammonium derivatives.

In the same way as on the standard strains [128], the contribution of the bis-heterocyclic functions does not lead to a significant gain in activity compared to the unsubstituted platforms, although the β-bipyridine and the bithiazole slightly increase the MIC values, particularly on INH-resistant strains. However, depending on the type of bis-heterocycle, the results differ, with an extinction of activity in the case of α-bipyridine: at a concentration of 10 µM, the inhibition goes from 76% to 0% for the ammonium derivatives and from 97 to 10% for the guanidiniums, while the inhibition activity remains the same with β-bipyridine.

**Scheme 5:**
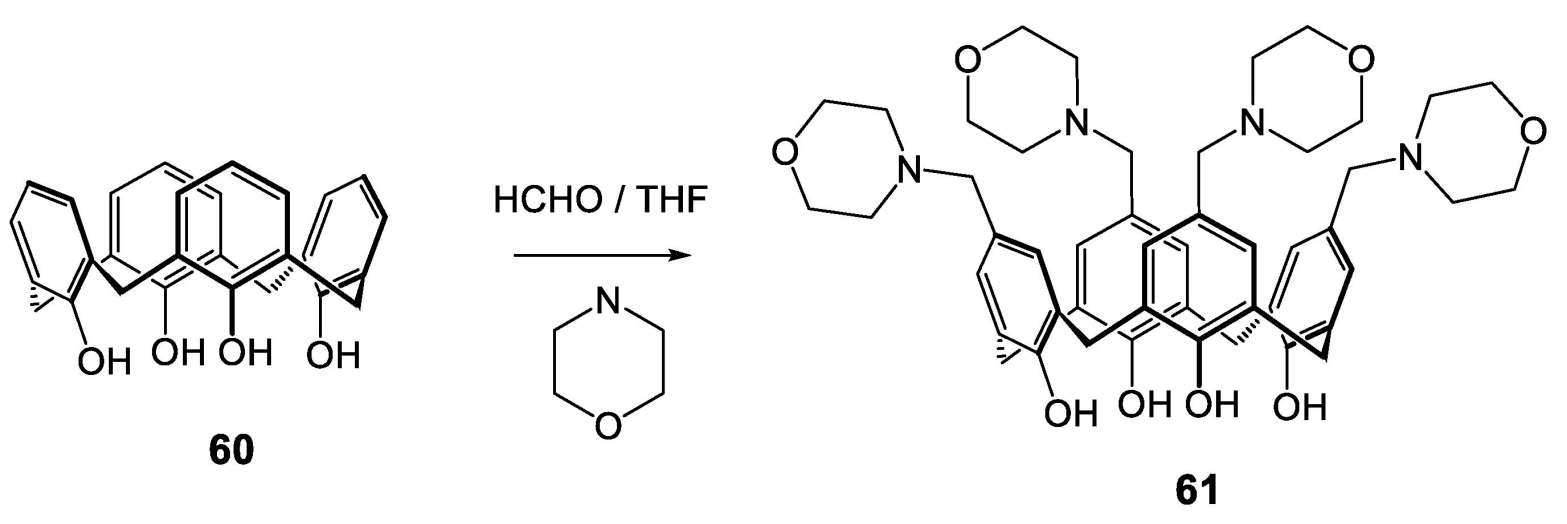
Synthetic pathway of *tetra*-morpholinocalix[4]arene **61** [133].

A water-soluble macromolecule based on calix[4]arene and morpholine units (**61** Scheme 5) was developed by Memon et al. in 2012 [133], which is easily obtained according to the Gutsche procedure [64] using detertiobutylated calix[4]arene, morpholine and formaldehyde via the Mannich reaction (Scheme 5). The antibacterial tests were carried out against two Gram-positive species: *Staphylococcus albus* and *Streptococcus viridians*, as well as five Gram-negative ones: *Bacillus procynous*, *Enterobacter aerogenes*, *Klebsiella aerogenous*, *E. coli,* and *Salmonella enterica* subsp. *enterica*. There appears to be an excellent activity profile for this *tetra*-morpholinocalix[4]arene. The inhibitions of growth were measured at 4 µg/mL against all strains, except for *Staphylococcus albus* and *Enterobacter aerogenes,* at 16 and 8 µg/mL, respectively.

**Scheme 6:**
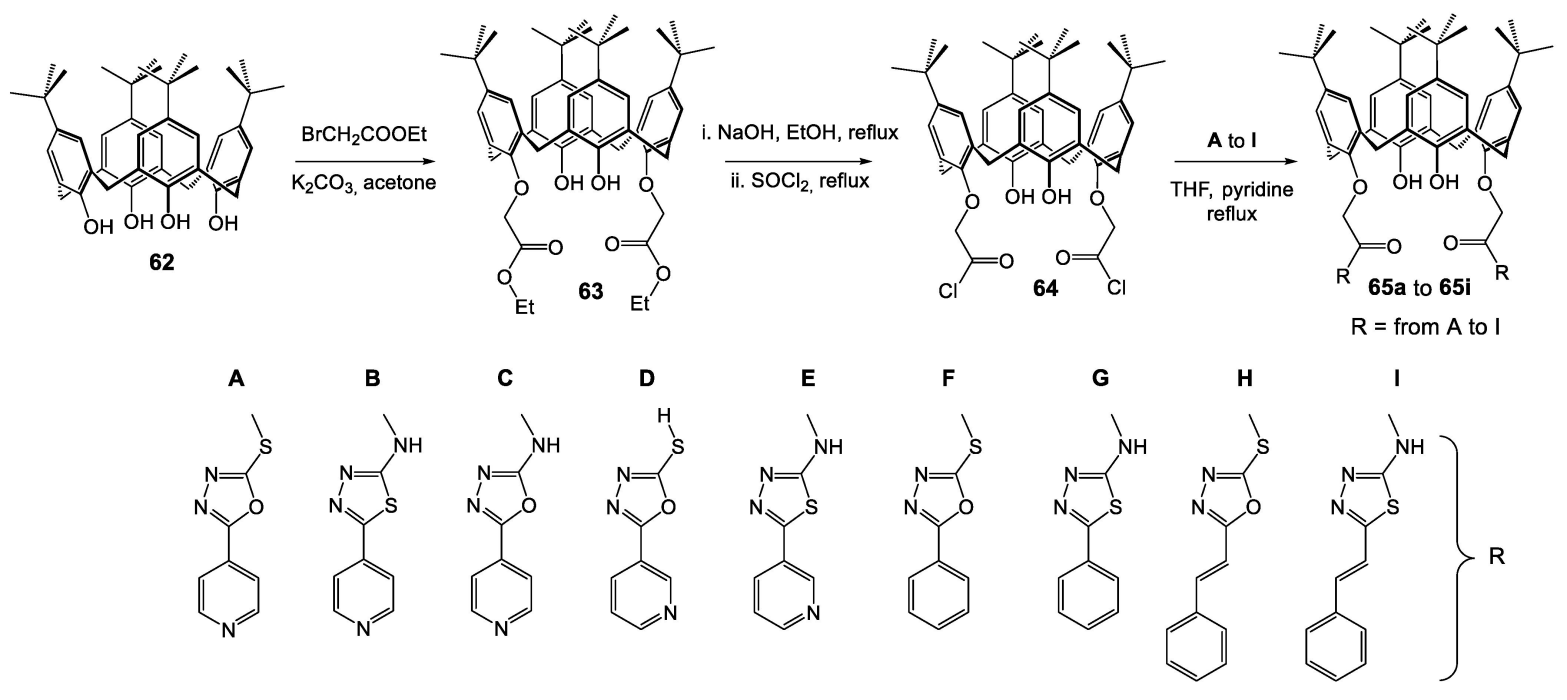
Synthetic pathway to compounds **65a** to **65i**, and representation of subunits 1,3,4-thiadiazole and 1,3,4-oxadiazole heterocycles **A** to **I** [134].

A series of nine new calixarenes based on 1,3,4-oxadiazole and thiadiazole were synthesized and have been subjected to antibacterial screening [134]. Based on the fact that the first-line anti-mycobacterium drug isoniazid (isonicotinic acid hydrazide: INH) is metabolized in the liver by acetylation and causes hepatotoxicity effects, the authors considered the replacement of hydrazide moieties of INH with 1,3,4-thiadiazole and 1,3,4-oxadiazole heterocycles, hoping to eliminate the in vivo acylation step. Therefore, they synthesized nine oxadiazole and thiazole derivatives of isoniazid, nicotinic, benzoic, and cis-cinnamic acid hydrazide **A** to **I** (Scheme 6). In parallel, the preparation of functionalized calixarene in the *1,3-alternate* position on the lower rim of bearing two ethanoyl chloride arms was performed from *tetra*-*tert*-butylcalix[4]arene **62**, via grafting of ester functions (**63** Scheme 6), saponification and final chlorination (**64** Scheme 6). Then oxa- and thia-diazole were introduced on **64** in THF in the presence of pyridine to give the expected derivatives **65a** to **65i** (Scheme 6).

Determination of MICs of the all calixarene derivatives **65a** to **65i** and the isoniazid derivatives with 1,3,4-thiadiazole and 1,3,4-oxadiazole heterocycles A to H were carried out against various Gram-negative (*E. coli* MTCC 443 and *P. aeruginosa* MTCC 1688) and Gram-positive (*S. aureus* MTCC 96 and *Streptococcus pyogenes* MTCC 442) bacteria strains, as well as against isoniazid-sensitive *Mycobacterium tuberculosis* (H_37_Rv). With regard to the other calixarene derivatives already described in literature, the antibacterial activities of these new compounds are moderate on Gram-positive and -negative strains. The MIC values fluctuate between 100 and 500 µg/mL. Also carried out on tubercular bacillus, this study gives a good to moderate bioactivity on the isoniazid-sensitive strain used (MIC values from 50 to 250 µg/mL). Better activities were measured for calixarenes **65a**, **65c**, and **62h** with MIC at 50, 62.5, and 62.5 µg/mL, respectively, compared with 0.2 µg/mL for isoniazid used as reference. All the values recorded do not make it possible to determine a notable difference between the different heterocycles introduced. However, in general, the substituted calixarenes seem to have an activity slightly greater than unsubstituted oxadiazole and thiadiazole (**A** to **I**); calixarene **65h**, for example, exhibits MIC at 100 µg/mL on *E. coli*, *P. aeruginosa,* and *S. aureus* compared with 250 µg/mL for the oxadiazole **H** subunit alone on the same strains. The authors justified these differences by increasing of the lipophilic character introduced via oxa- or thia-diaozole and calixarenic core and facilitating the crossing through of the biological membrane. A cooperative effect of the pharmacophore is also advanced to justify the enhancement of activity of substituted calixarenes (**65a**–**65i**) compared to unsubstituted oxadiazole and thiadiazole (**A**–**I**).

**Scheme 7:**
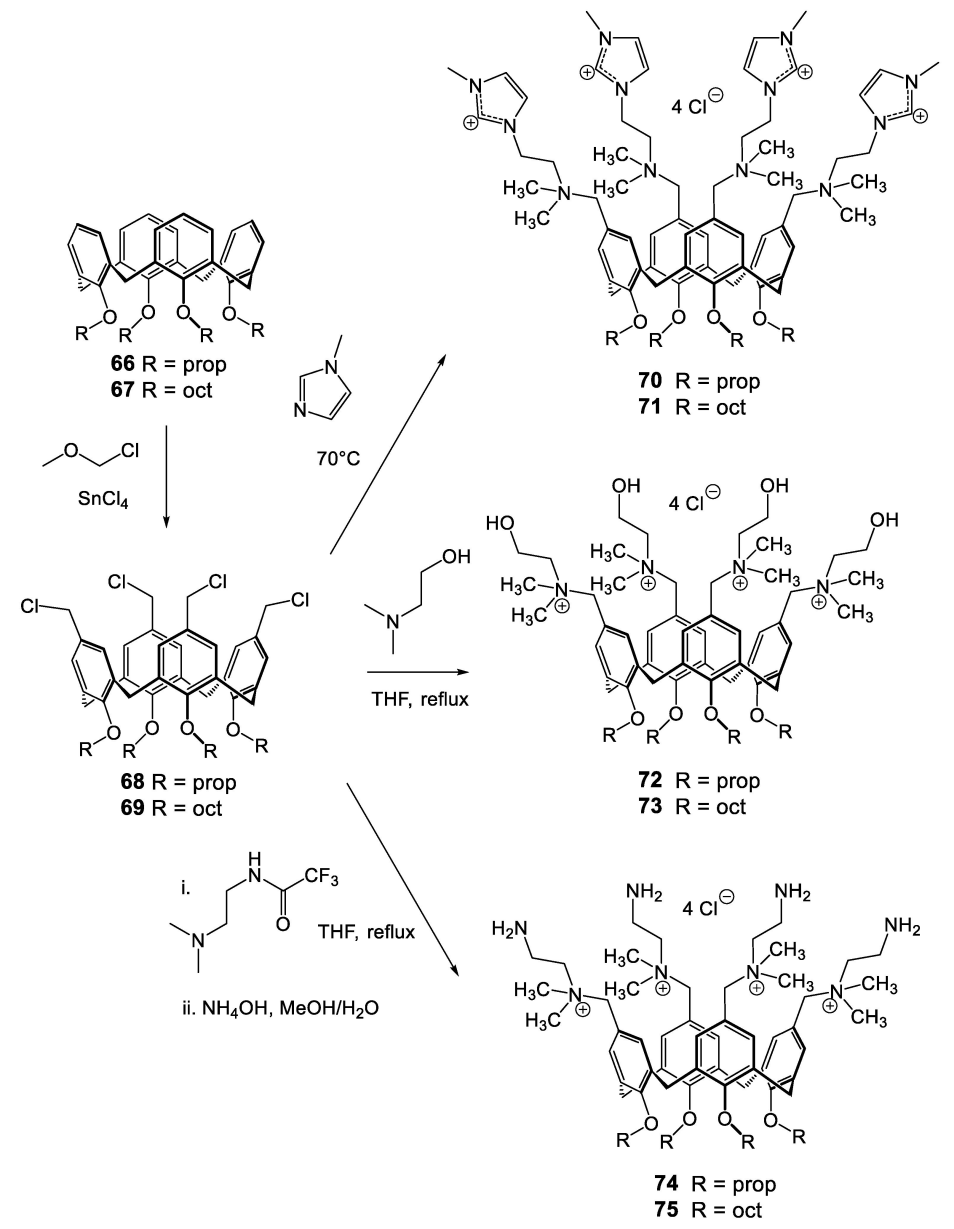
Synthetic pathway to cationic calixarenes modified with methylimidazolium (**70**, **71**), choline (**72**, **73**) and ethylenediamine (**74**, **75**) groups [135]. prop: propyl, oct: octyl.

In 2015, Melezhyk et al. published the antibacterial properties of *tetra*-alkylammonium and imidazolium calix[4]arene derivatives [135] compared to macrobicyclic peptidocalix[4]arenes **18** and **19** (Scheme 1) [115], trifluoroacetate of *tetra*-guanidinium calix[4]arene **28** (Scheme 2) [72], and morpholinocalix[4]arene **61** (Scheme 5) [133]. These new cationic structures were obtained from *tetra*-propyloxy- or *tetra*-octyloxy-calix[4]arene (**66** or **67**) chloromethylated with couple methyl chloromethyl ether/tin *tetra*-chloride (intermediates **68** and **69** Scheme 7), then chlorine displacement by *N*-methylimidazole or *N*,*N*-dimethylethanolamine (choline) to give **70**–**71** and **72**–**73**, respectively, or by trifluoroacetamide of *N*,*N*-dimethylethylendiamine followed by treatment in ammonia solution, leading to **74**–**75** (Scheme 7).

The antibacterial activities were determined by MIC measurement on *P. aeruginosa* ATCC 9027, *S. aureus* ATCC 6538, *K. pneumoniae* ATCC 10031, and *E. coli* ATCC 25922 strains by the broth microdilution method. The first point is the significant decrease in activity with the switch from the propyl derivative (10 to 1000 µg/mL) to the octyl derivative (500 to 1000 µg/mL), i.e., 5 to >100 times less activity for the *tetra*-octylcalixarenes (Table 6). The authors propose a possible explanation for the lower antibacterial activity of long-chain derivatives based on their stronger self-assembly ability in water solution (Critical micellar concentrations at 3 × 10^−6^ M and 8 × 10^−5^ M for octyl **73** and propyl **72**, respectively) [136].

In addition, there is clearly a difference in activity between the cations studied, with a better activity of the imidazole derivative against all strains tested (MIC < 10 µg/mL on *E. coli*, *S. aureus* and *K. pneumoniae,* and 50 µg/mL on *P. aeruginosa*). The choline and ethylenediamine exhibit higher MICs between 10 and 1000 µg/mL according to the strains. The differences observed were attributed to the mode of interaction with bacterial membrane structures according to the nature of cationic centers, and were also potentially linked to the distance between two opposite-site nitrogen atoms, an important value to optimize the interactions with the negatively charged groups of the bacterial membrane. Moreover, The MIC values are close to those of antibiotics and better or equal to those of quaternary ammonium salt used as reference. For example, the imidazole derivative **70** exhibits a MIC <10 µg/mL against *K. pneumoniae*, while tetracycline, vancomycin, and didecyldimethylammonium chloride are measured at 16, 64, and 16 µg/mL respectively.

In addition, there is clearly a difference in activity between the cations studied, with a better activity of the imidazole derivative against all strains tested (MIC < 10 µg/mL on *E. coli*, *S. aureus* and *K. pneumoniae,* and 50 µg/mL on *P. aeruginosa*). The choline and ethylenediamine exhibit higher MICs between 10 and 1000 µg/mL according to the strains. The differences observed were attributed to the mode of interaction with bacterial membrane structures according to the nature of cationic centers, and were also potentially linked to the distance between two opposite-site nitrogen atoms, an important value to optimize the interactions with the negatively charged groups of the bacterial membrane. Moreover, The MIC values are close to those of antibiotics and better or equal to those of quaternary ammonium salt used as reference. For example, the imidazole derivative **70** exhibits a MIC <10 µg/mL against *K. pneumoniae*, while tetracycline, vancomycin, and didecyldimethylammonium chloride are measured at 16, 64, and 16 µg/mL respectively.

**Scheme 8:**
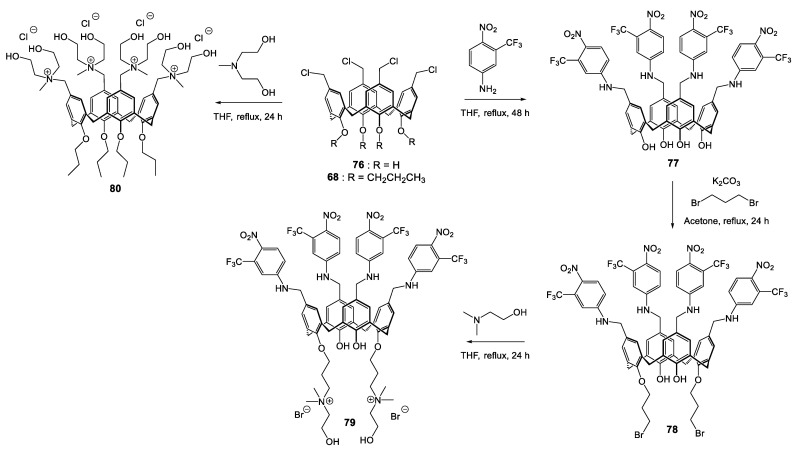
Synthetic route for the preparation of polycationic calixarenes **79** and **80** [137,140].

In 2017, Consoli et al. [137] used the calixarene platform to cluster multiple NO photodonor units, associated on the other side of platform at two quaternary ammonium groups to introduce a solubility in hydro-alcoholic solvent and allow an electrostatic interaction with the bacteria wall according to Scheme 8. Treatment of chloromethylated calix[4]arene (**76** Scheme 8) by 3-(trifluoromethyl)-4-nitrobenzenamine gave the intermediate **77**, on which the introduction of quaternary ammonium moieties was carried out in two steps in the *1,3-alternate* positions of the lower rim: addition of dibromopropane in K_2_CO_3_ medium (intermediate **78**) followed by substitution of residual halogens by *N*,*N*-dimethyl-ethanol amine to give the expected ammonium **79**. The investigations of antibacterial effects were conducted on *S. aureus* ATCC 6538 and *E. coli* ATCC 10536 strains. For the NO-donor derivative **79**, a reduction of 98.9% of bacterial load appears after 30 min in dark conditions (reduction of 1.96-log in CFU/mL), and under irradiation, the effect was greater, with a reduction of 99.2% (−2.1-log) at 10 min and up to 99.95% (−3.31-log) after 20 min.

On the other hand, the impact on *E. coli* is different, with non-activity detected in dark conditions and a reduction of bacterial load of only 93.5% (minus 93.5-log) after 30 min of irradiation. The understanding of these activities comes, according to the authors, firstly by the presence of quaternary ammonium functions, which may bind the negatively charged bacterial wall by electrostatic interactions, and also by the hydroxylamines grafted on the polar head, known for their capacity to penetrate the bacterial membrane [138]. This first point explains the activity observed in dark conditions for *S. aureus*, the Gram-negative strain (*E. coli*) being more resistant to quaternary ammonium salts [139]. The increase in bacteria load under irradiation is, meanwhile, associated with the light-controlled NO-releasing.

In a second study, the same authors described the synthesis of an another polycationic calix[4]arene bearing four ethanol ammonium groups in the upper rim (**80** Scheme 8) [140].

Treatment of the *tetra*-propoxy-chloromethylcalix[4]arene derivative **68** with *N*-methyldiethanolamine allows the formation of *tetra* quaternary ammonium salt **80** (Scheme 8). In the same way as derivative **79** (Scheme 8), this new cationic compound exhibits an excellent activity at 4.0 µg/mL against Gram-positive *S. aureus* reference strain ATCC 6538 and methicillin-resistant strain (MRSA 15) as well as on *S. epidermidis* (reference ATCC 35984 and methicillin-resistant MRSE 57 strains). The Gram-negative strain is less sensitive to the ammonium salt, where the active concentration reaches 128 and 256 µg/mL on *P. aeruginosa* reference strain ATCC 9027 and the antibiotic-resistant clinical isolate, respectively. Analogously to the cationic calixarenes previously described [72,120] the authors advance a perturbation of the cell membrane via electrostatic interactions associated with hydrogen bonds and hydrophobic interactions in which the OH groups and the alkylated aromatic core could be involved. Moreover, the derivative **80** was much more efficient on MRSA 15 and MRSE 57 than ofloxacin or chloramphenicol, with MICs 32-fold to 128-fold lower.

This Italian team also started, through this study, an evaluation of the antibacterial activity of this calixarene in synergy with various known antibacterial compounds and with the objective of detecting additivity or synergy. Thus, the strains presented above made it possible to evaluate in vitro the additive, synergistic, or antagonistic capacities of calixarene **80** in association with ofloxacin, chloramphenicol, or tetracycline.

The results showed that although none of the calixarene **80**/antibiotic combinations bring a synergistic effect, there is also no antagonism. If the effects were mostly indifferent, it appears an interesting additivity against *P. aeruginosa* isolate 1, allowing to record a reduction of initial MICs by a factor 2, 8, 32, and 16 of calixarene **80**, ofloxacin, chloramphenicol, and tetracycline, respectively. The authors consider this additive effect by a possible enhancement of membrane permeability generated by the cationic calixarene, allowing a better uptake of antibiotic through the bacterial wall, as described by Formosa et al. [124]. These results, in accordance with the observations of Grare and Duval [125], and the synergistic effect on *P. aeruginosa* via association with a cationic calixarene, are very encouraging for the development of new strategies to fight the bacterial resistance of this priority pathogenic strain.

**Figure 9:**
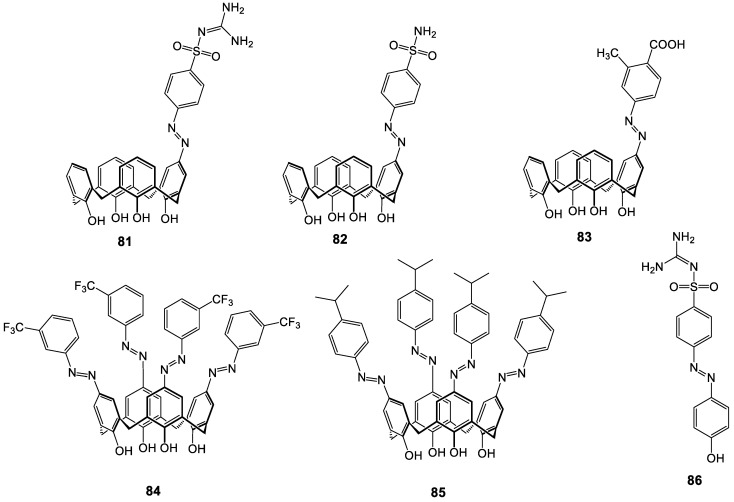
Mono-azo **81**, **82**, **83**, tetra-azo **84**, **85** calixarene derivatives and monomer **86** [141].

While a number of azo groups exhibit various therapeutic activities, their introduction could increase the antibacterial activities of certain molecules; their use in this sense, via the calixarenic core; has not been evaluated. On these observations, Ali and co-workers synthesize a series of mono- and tetra-azo calixarene derivatives [141]. These new structures presented in Figure 9 were synthesized according to the reported procedure [142,143] with slight modifications, such as the molar ratio of reactants and reaction times for obtaining notably mono-azo derivatives.

The synthesized compounds were screened against five Gram-positive bacterial strains (*B. subtilis*, *S. aureus*, methicillin-resistant *Staphylococcus aureus* (MRSA), *S. epidermidis,* and *E. faecalis*), and two Gram-negative bacterial strains (*E. coli* and *P. aeruginosa*). The results, shown in Table 7, are clearly in favor of an activity on Gram-positive strains. Azo-based calixarene **82** showed an excellent activity, the best among the compounds tested. It exhibits a range of MICs between 0.97 μg/mL on MRSA and *B. subtilis* and 7.8 μg/mL on *E. faecalis* regarding Gram-positive bacteria. These values are equivalent to or better than those measured on Gentamycin used as the reference drug. The activity of **82** may be attributed to the presence of sulfonamide units known for their antibacterial action. The derivative sulfaguanidine-based **81** is also very active on Gram-positive strains, with MIC values at 7.8 µg/mL on *S. aureus* and *S. epidermidis* or 15.6 µg/mL on MRSA and *B. subtilis*, but contrary to the **82** analogue, it is also active against *P. aeruginosa* (15.6 µg/mL). Activities of derivative **81** were compared to those of its constitutive monomer **86**, which is inactive on all the strains tested, indicating that the activity of azo-based calixarene could be due to the combined effect of a guanidinium unit macrocyclic ring, similar to that reported by Mourer et al. [72] for antibacterial evaluations, but also by de Fátima and co-authors [144] in research on the antifungal properties of functionalized calixarene. The benzoic acid derivative **83** also has a remarkable activity close to those determined with the **82** analogue, although the authors expected a better activity due to its resemblance with *p*-aminobenzoic acid, which is produced by bacterial cells for biosynthesis of folic acid, essential to its growth. The *tetra*-substituted calixarenes **84** and **85** are, respectively, non- to moderately active, greater than 126 μg/mL for **84** and at best 15.6 and 31.25 μg/mL for **85** against *E. faecalis* and MRSA, respectively. Moreover, molecular docking was performed to see the interaction of the designed compounds with penicillin-binding protein receptors (extracellular membrane-bound enzymes); inhibition of it led to cell death. It appears that the results obtained are correlated with the inhibitory capacity measured in vitro. The most affinity was measured for the two derivatives **81** and **82**, followed by **83**, **86**, **85,** and **84**.

In 2019, Mourer et al., still based on a hypothesis of a mechanism of action involving deleterious electrostatic interactions between negative bacterial surface charges and the charges introduced on a spatial organizer such as calixarene, described the first study comparing the antibacterial activity of the different conformers of a cationic calixarene derivative [103].

**Scheme 9:**
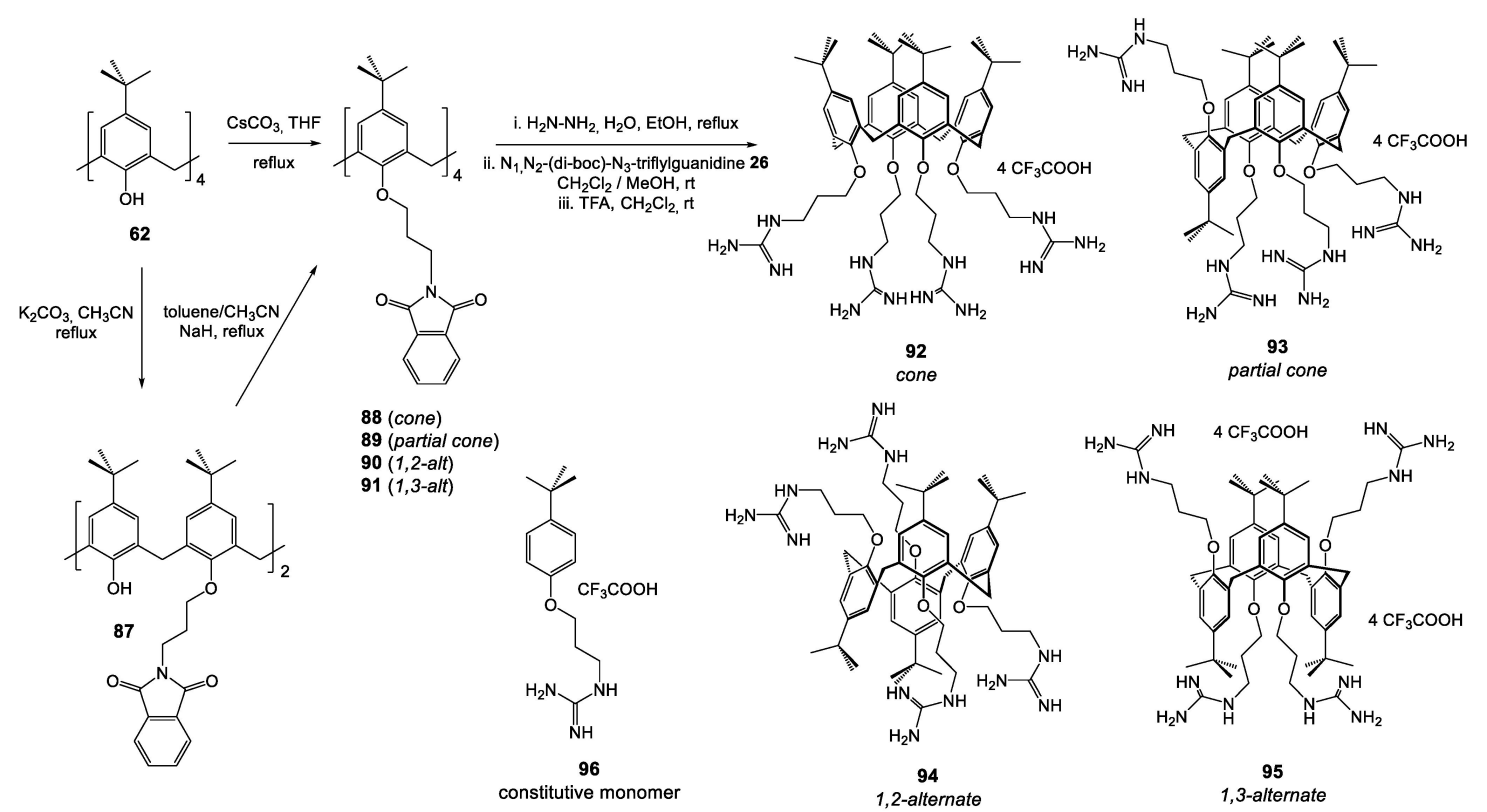
Synthetic pathway to four conformer guanidinium derivatives **92**, **93**, **94**, **95** and structure of monomeric analogue **96** [103].

The four conformers were obtained in four steps from *para*-*tert*-butylcalix[4]arene **62** according to a procedure adapted from Böhmer and coworkers [145] (Scheme 9). Treatment of **62** with *N*-(3-bromopropyl)phthalimide in presence of cesium carbonate led, after treatment by crystallization and chromatography, to the three conformers *partial cone* **89**, *1,2-alternate* **90**, and *1,3-alternate* **91**. The cone conformer analogue **88** was obtained in two steps with a first bis-alkylation by *N*-(3-bromopropyl)phthalimide of **62** in K_2_CO_3_ medium (**87** Scheme 9), following by alkylation at the residual OH functions by treatment with *N*-(3-bromopropyl)phthalimide in the presence of NaH as base. The phthalimide cleavage process by a standard procedure with hydrazine was followed by introduction of guanidine moieties with the *N*_1_,*N*_2_-(di-Boc)-*N*_3_-triflylguanidine **26**. Then, a final deprotection in acidic medium afforded the expected *tetra*-guanidinium conformers **92** to **95**.

These four calixarenic conformers **92** to **95** and the constitutive monomer **96** were evaluated for their in vitro antibacterial activities against Gram-positive (*E. faecalis* ATCC 29212 and *S. aureus* ATCC 25923 and ATCC 29213) and Gram-negative (*E. coli* ATCC 25922 and *P. aeruginosa* ATCC 27853) bacteria, as well as INH-sensitive (H_37_Rv) and -resistant (MYC5165) *Mycobacterium tuberculosis* (Table 8).

The amphiphilic conical compound **92** displays the lowest activity, with a MIC of 8 to 128 µg/mL on standard strains and 78 µg/mL on *M. tuberculosis*, but nevertheless exhibits low toxicity (approximately 140 µg/mL on the noncancerous human pulmonary embryonic fibroblast (MRC-5) cell line). A significant increase in activity on all the strains appears from the reversal of a single cycle (*partial cone* **93**) of the calixarenic crown, for example, MICs of 128 to 8 µg/mL on *E. coli*, respectively, for the *cone* **92** and *partial cone* **93**, but also a MIC divided by 4 on INH-resistant *M. tuberculosis* for the same compounds. The switch to the *1,3-alternate* bolaamphiphile conformation **95** generated the most enhancement of antibacterial activities of the series, in particular on *M. tuberculosis*, with 5 and 0.8 µg/mL on H_37_Rv and MYC5165, respectively.

In terms of toxicity, it appears that the cycle reversal in the case of the *partial cone* **93** and *1,2-alternate* **94** derivatives results in the appearance of a certain toxicity 2 to 2.5 times greater respectively than in the conical derivative. On the other hand, the *1,3-alternate* derivative **95** appears to be the least toxic of the series (>256 µg/mL) for excellent selectivity indexes.

The authors attribute these differences in behavior to the orientations of the different hydrophilic (guanidine part) and lipophilic (aromatic nuclei and propyl ether components) regions, modifying the amphiphilic natures of these calixarene species, from normal amphiphilicity for **92** to bolaamphiphilicity, partial for **93**, and full for **94** and **95**. The variation of the hydrophilic/hydrophobic balance of various functional groups grafted on the calixarenic core and their organization as bolaamphiphiles appears to modify the in vitro activity. Moreover, the *1,3-alternate* conformer, with the highest symmetry and alternating guanidinium *para-tert*-butyl groups on each rim of the calixarene crown, seems to represent the best compromise for good activity and low toxicity.

1,3,4-oxadiazole nucleus and its derivatives have attracted much attention because of their pharmaceutical applications, such as antimicrobial, antituberculostatic, or fungicidal agents. In 2020, Gezelbash and Dilmaghani used the oxadiazole core, as bioisosteres of amides and esters, and coupled them to the calixarene skeleton to generate synergy in the therapeutic effect [146].

**Figure 10:**
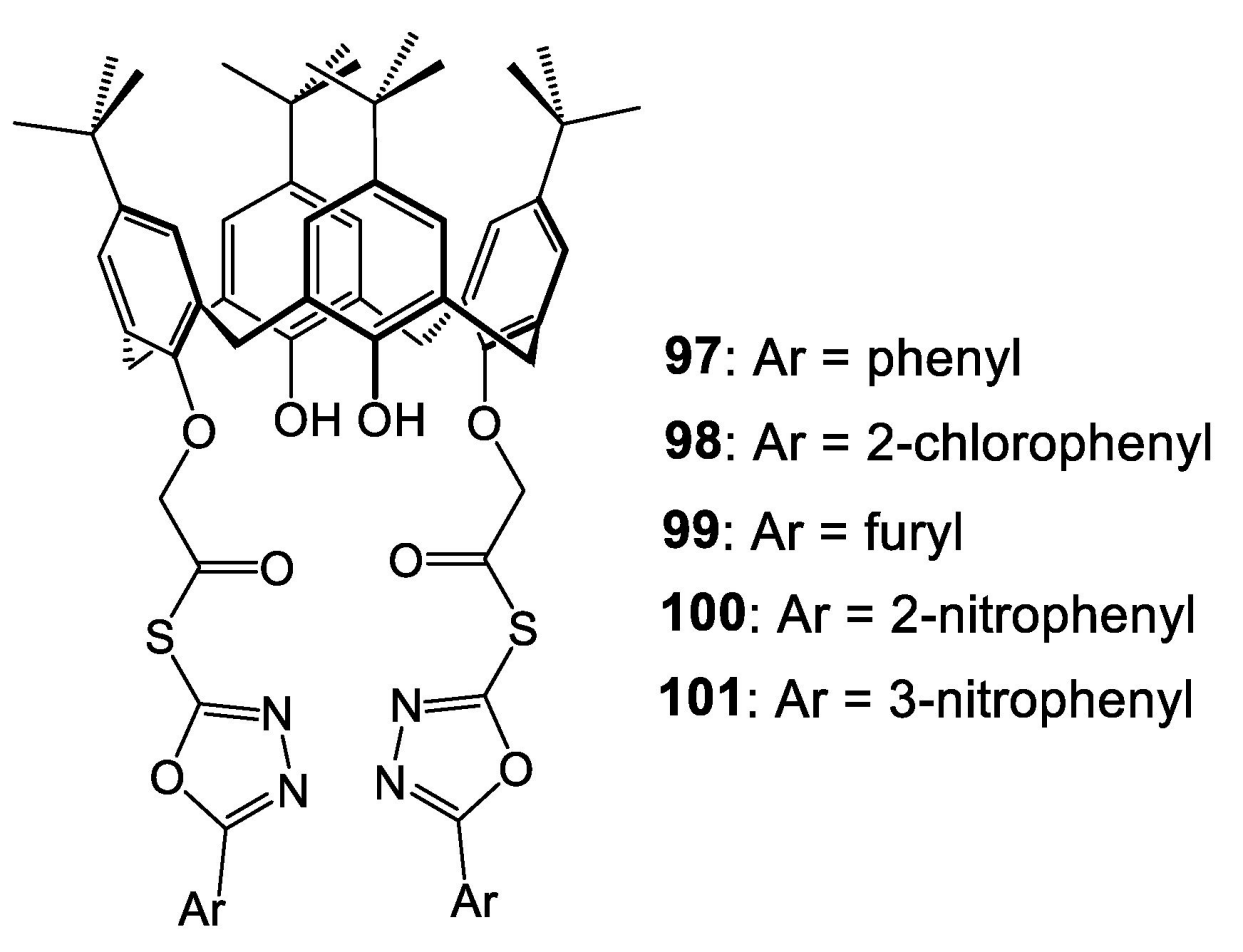
Structure of oxadiazole-based calixarenes **97** to **101** [146].

The new oxadiazole-based calixarenes **97** to **101** presented in Figure 10 were obtained by reaction of *para*-*tert*-butylcalix[4]arene **62** with the corresponding 1,3,4-oxadiazole acyl chloride derivatives under carbonate medium in DMF. The antibacterial evaluation was carried out against the *E. coli* strain. Unfortunately, the results do not seem convincing for a synergistic effect provided by the calixarenic platform. No activities were recorded for calixarenes **97** and **98** compared with the corresponding 1,3,4-oxadiazole acyl chlorides, with MICs at 10 and 15 µg/mL, respectively. Only **97** and **101** presented an antibacterial activity where the initial oxadiazole acyl chloride was inactive. However, the authors provide no explanation for the measurements acquired.

A very recent study complements previous work on positively charged antibacterial structures (ammonium, imidazolium, guanidinium). In 2021, Padnya et al. have developed a series of thiacalix[4]arene-based quaternary ammonium functions under both *cone* and *1,3-alternate* conformations [147].

**Scheme 10:**
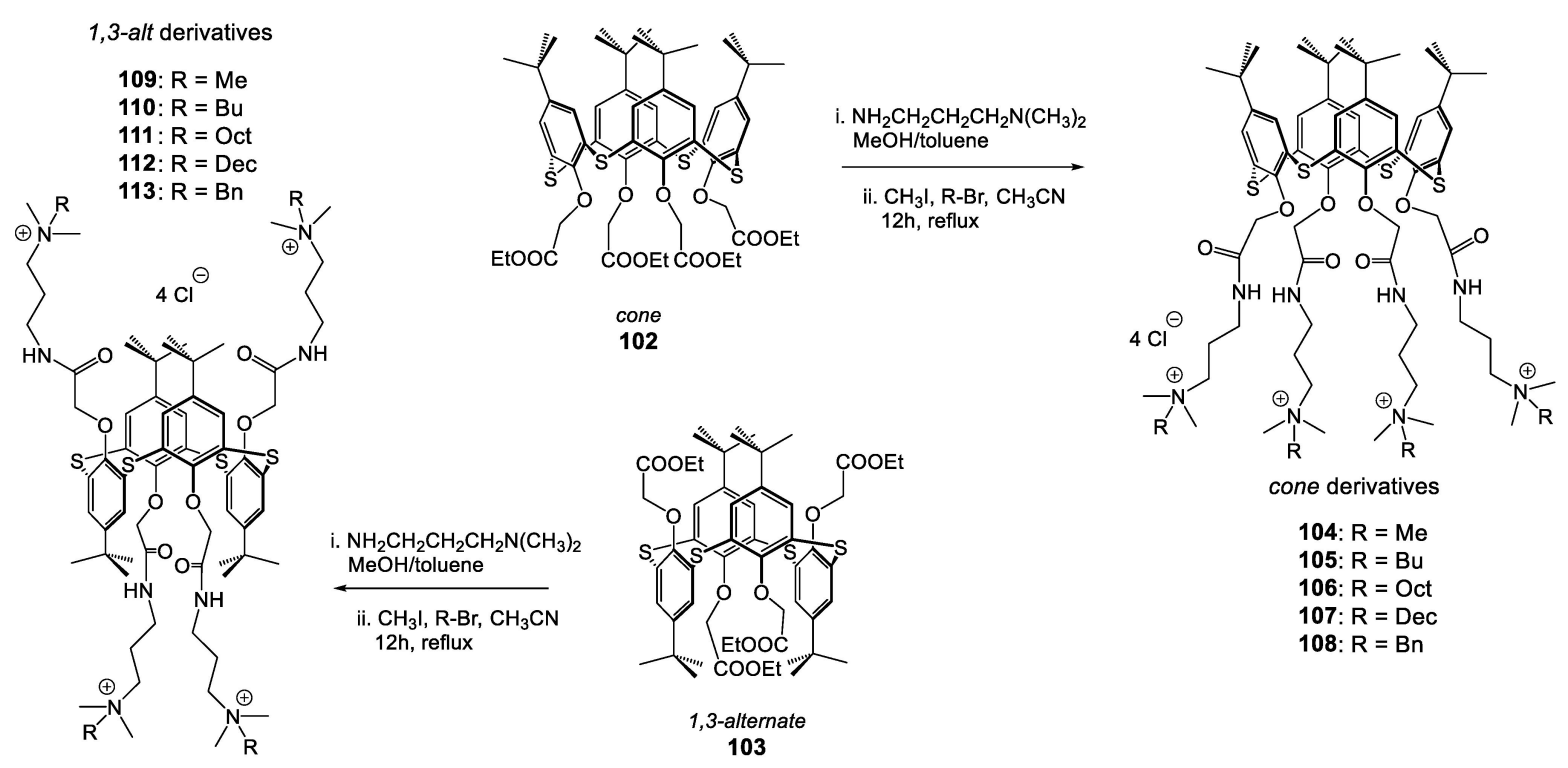
Synthetic route for the preparation of quaternary ammonium salts of thiacalix[4]arene **104** to **113** [147]. Me: methyl, Bu: butyl, Oct: octyl, Dec: decyl, Bn: benzyl.

From **102** in cone conformation, or from its *1,3-alternate* analogue **103** and according to the same operating conditions, they obtain, in two steps, the ammonium compounds **104** to **108** (conical derivatives) and **109** to **113** (*1,3-alt* derivatives) with excellent yields (>90%). Ester derivatives are easily converted to amides by the introduction of *N*,*N*-dimethylaminopropylamine. In a second step, the tertiary amines are alkylated by various halogenated derivatives (bromide or iodide). Finally, the passage on an ion exchange chromatography makes it possible to obtain the 10 new quaternary ammonium compounds in the form of chloride salts (Scheme 10).

MIC and MBC measurements were conducted on three Gram-positive strains (*S. aureus* ATCC 29213, a clinical isolate of *S. epidermidis,* and *B. subtilis* 168) as well as three Gram-negative strains (*E. coli* ATCC 25922, *K. pneumoniae* 1813, and *P. aeruginosa* ATCC 27853). The results are presented in Table 9 and have been compared, among others, with the activity of chlorhexidine used as a reference. Except for the **109** derivative, all the compounds studied have a very good antibacterial activity, with MIC values often below 16 μg/mL, and close to the activities measured with reference compounds. As previously observed [103], the results show that the derivatives in the *1,3-alt* conformation exhibit higher activities compared with the conical homologues. The *1,3-alt* octyl thiacalix[4]arene **111** produces the lowest MIC values of 1 to 2 µg/mL and 8 to 32 µg/mL, respectively, against Gram-positive and Gram-negative strains.

The authors also measured the cytotoxic activities of these thiacalixarene-based quaternary ammonium salts. The cell viability measured on human skin fibroblast cells allows the calculation of the selectivity index. In agreement with the work of Mourer et al. [103], there clearly appears to be lower toxicity for *1,3-alternated* conformers, with CC_50_ values close to 1000 µg/mL for **109**, **111**, and **113**. Based on the selectivity index values, the study shows that the most promising antibacterial candidate is the 1,3-*alt*-Oct derivative **111** against both Gram-positive and Gram-negative bacteria strains. To complete the study, the authors tested three most active candidates against 16 Gram-positive and Gram-negative strains from clinical isolates. In the same way as on standard strains, the compound **111** exhibits the weakest MIC values, more particularly on Gram-positive strains.

Finally, to complete the study and try to understand the activities measured, the authors evaluated the different types of interactions of the *cone*-Oct **106** and 1,3-*alt*-Oct **111** derivatives with two lipid membrane models: DPPC (1,2-dihexadecanoyl-*sn*-glycero-3-phosphocoline) and POPG (1-palmitoyl-2-oleyl-*sn*-glycro-3-phospho-(1′-*rac*-glycerol). The evolution of the hydrodynamic diameters, of the polydispersity indexes, and of the zeta potentials of the vesicles in the presence of thiacalixarene indicates different modes of interaction. The conical compound is adsorbed on the surface of the vesicles, while the *1,3-alt* derivative leads to clumping of these. These two different interaction mechanisms could explain the variation in activity of the two conformers. As proposed in previous studies of other groups [62,115,119], the authors suggest a mechanism of antibacterial action, based initially on an approach of thiacalixarene generated by electrostatic interactions (negatively charged bacterial membrane), followed by a membrane integration of the hydrophobic parts.

**Scheme 11:**
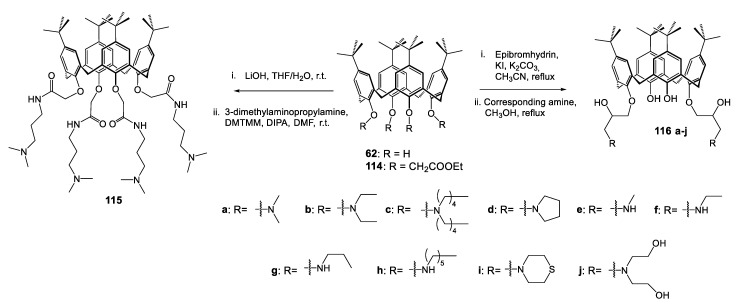
Synthetic way to obtain new cationic amphiphilic calix[4]arene derivatives **115** and **116a-j** [148]. DMTMM: 4-(4,6-dimethoxy-1,3,5-triazin-2-yl)-4-methyl-morpholinium chloride, DIPA: diisopropylamine.

In 2022, Fang et al. designed and synthesized a series of bis- and tetra-functionalized calix[4]arenes as membrane-active antibacterial agents. Synthesized from a *tertio*butylated calix[4]arene platform (from **62** or **114**), they introduce either two or four amino functions into the lower rim (Scheme 11) [148]. The latter become potentially cationic at physiological pH and can thus exert their action. As hypothesized in previous studies [62,115,119], these cationic amphiphilic structures will again be able to interact electrostatically with the negatively charged bacterial membrane. Here again, the authors point out that the lipophilic charge on the upper rim (*tertio*-butyl group) could facilitate the insertion of the structure into the phospholipid bilayer of the membrane, altering the permeability and causing a deleterious effect on the cell.

Tested on the Gram-positive reference strain *S. aureus* ATCC 29213 and two resistant strains MRSA N315 and MRSA NCTC10442, the results are very promising, especially for the *tetra*-functionalized compound **115** incorporating 3-dimethylaminopropylamine functions. Indeed, it shows minimal inhibitory concentrations of 1.6, 1.6, and 3.1 µg/mL, respectively, on the three strains mentioned above. In addition, the authors showed a rapid action of the compound **115**, where the time–kill kinetics curves against MRSA NCTC10442 show a decrease of 4.3 to 5.6 log of the bacterial population in 0.5 h to, respectively, 4 and 8 times the MIC. In comparison, vancomycin, used as a reference, only leads to a reduction of 2 log to 8 times its CMI in 4 h.

The membrane target and increased permeability were supported by the use of the SYTOX Green dye, which easily penetrates bacteria with damaged membranes and for which the fluorescence intensity increases when bound to intracellular nucleic acids. The increase in **115**-concentration on MRSA NCTC10442 is indeed accompanied by a very significant increase in fluorescence intensity.

### 7.2. Molecular Carrier/Drug Delivery

Widely used in the search for new active compounds, the principle of carriers is part of the strategies for the development of new active ingredients. In the case of calixarenes, the authors generally seek to use the platform in order to modify the bioavailability (increase the hydro- or the lipo-solubility for examples), increasing the activity of the active ingredient by generating a greater density of antibiotic per unit surface (cluster effect), and this via strong covalent bonds or physiologically hydrolyzable bonds.

**Scheme 12:**
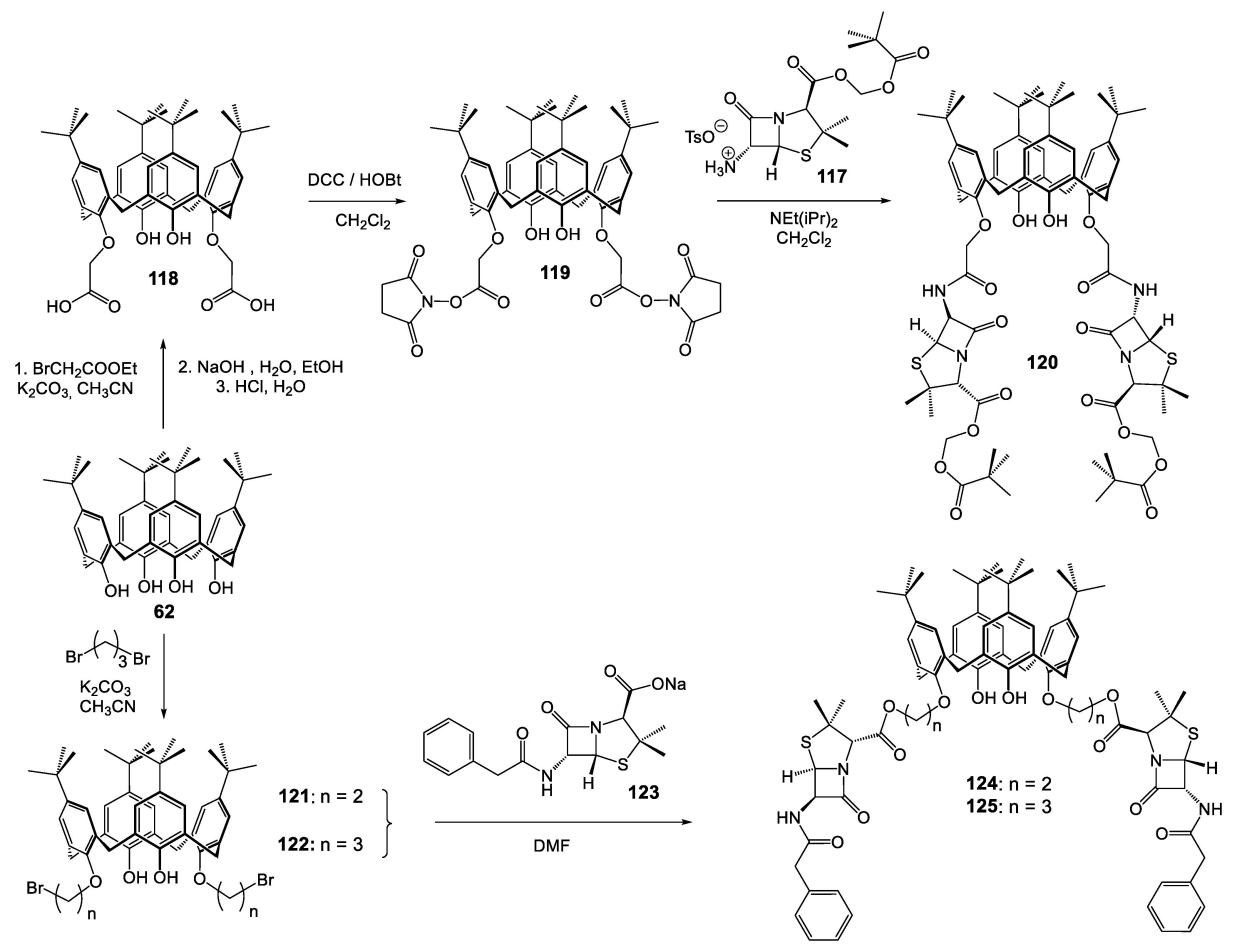
Synthetic way to obtain the penicillinic podands **120**, **124**, and **125** [149,150].

In 2001, Regnouf-de-Vains and Ben Salem initiated a study to develop calixarene-based podands shaped as potent drug dispensers, first with antibiotic penicillin grafted in alternate positions of the cone conformer of the *para*-*tert*-butylcalix[4]arene [149]. The synthetic pathway described in Scheme 12 involved the formation of hemi-synthetic penicillin via the formation of an amide bond. Penicillin was firstly protected at the carboxy group by a pivaloyloxymethyl ester function (**117** Scheme 12), thus giving a lipophilic prodrug which should release the biologically active free acid after hydrolysis by esterases, a peptide coupling via the formation of bis-activated ester **119** between the diacid calixarene **118**, *N*-hydroxysuccinimide and dicyclohexylcarbodiimide (Scheme 12). Reaction between the penicillin salt derivatives **117** and **119** in mild conditions gave the expected bis-penicillin podand **120**.

The authors also developed a *para*-*tert*-butylcalix[4]arene grafted by two penicillin G arms at the lower rim via ester linkage [150]. The choice of *para*-*tert*-butylcalixarene was directly related to a possible application of the molecules studied as prodrugs. An oral administration would lead to the release of the soluble antibacterial agent in the intestinal compartment; the insoluble, nontoxic calixarene molecule could be thus easily eliminated from the organism. The 1,3-bis(bromoethyl)- **121** or 1,3-bis(bromopropyl)-calix[4]arene **122** react in dry DMF with penicillin G sodium salt **113** to give **124** (47%) and **125** (32%), respectively (Scheme 12).

The amphiphilic structures 6-pivAPA **120**, and two benzylpenicillin derivatives **124** and **125**, were the subject of preliminary study of the possibility of incorporation and/or translocation across a model bacterial membrane lipid, using lipid monolayers prepared from 1,2-dimyristoyl-sn-glycero-3-phosphoethanolamine (DMPE) [150]. The investigations, assessed by surface pressure and surface potential measurements, as well as Brewster angle microscopy, show a possible incorporation of benzylpenicillin derivatives **124** and **125** into the biological membrane whereas the 6-pivAPA **120** could be more easily translocated across the membrane, probably due to the aliphatic pivaloyl terminal moieties decreasing molecule packing in the monolayer compared to the benzyl moieties.

**Scheme 13:**
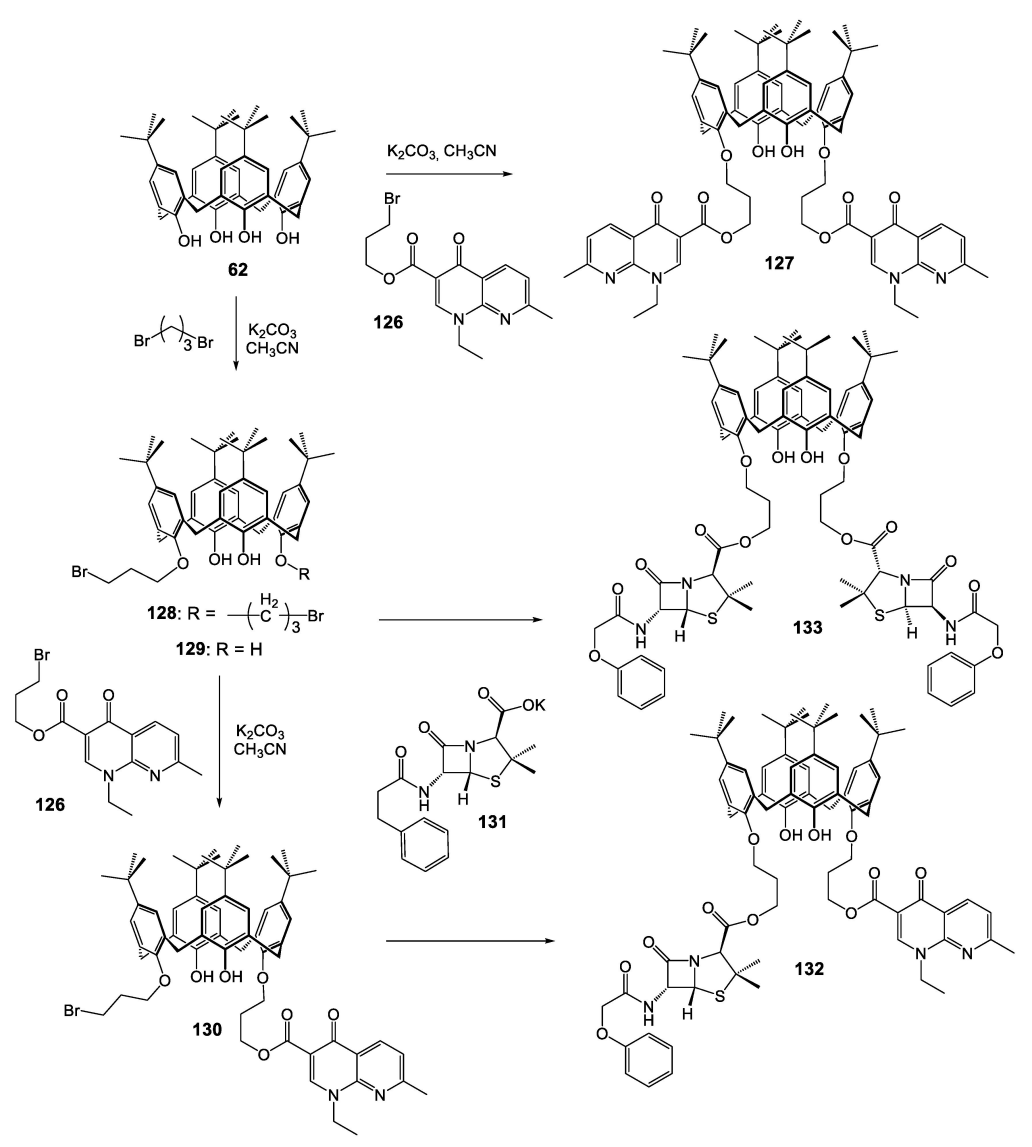
Synthetic pathway leading to the dissymmetric podand (penicillin V and nalidixic acid) **132**, the corresponding bis-nalidixic **127**, and bis-penicillin V **133** [151,152,153].

The same strategy involving ester linkage allows the synthesis of bis-quinolone derivative **127** [151,152]. The bromopropyl ester of nalidixic acid **126** was initially prepared by reaction in DMF of an excess of 1,3-dibromopropane with sodium salt of nalidixic acid (Scheme 13). Then the reaction with *para*-*tert*-butylcalix[4]arene **62** was performed in basic conditions using K_2_CO_3_ as base to finally afford the bis-nalidixic podand **127** with an overall yield of 35%.

Using the monobromopropylcalix[4]arene **129** as starting material and bromopropyl ester of nalidixic acid **127** in the presence of 0.6 equivalent of K_2_CO_3_ led the authors to obtain the mononalidixic derivative **130**. Incorporation of penicillin V onto the residual brominated arm was performed in dry DMF at 35 °C, under an inert atmosphere, by reacting **130** with an excess of potassium salt of penicillin V (PVK) **131** to give the nalidixic acid/penicillin V calix[4]arene podand **132** (Scheme 13) [153] that could mix synergistically two antiviral strategies, inhibition of AND-gyrase with quinolone and inhibition of peptidoglycan reticulation with penicillin. The 1,3-bis-propylpenicillinate **133** was prepared in 32% yield by direct reaction of **131** on the 1,3-bis-(3-bromopropyl)-calixarene **128** in dry DMF (Scheme 13).

The first antibacterial evaluations were performed with disk diffusion assays in DMSO solution of the dissymmetric podand (penicillin V and nalidixic acid) **132,** the corresponding bis-nalidixic **127**, and the bis-penicillin V **133** [153], against three Gram-positive reference strains, *S. aureus* ATCC 25923, *S. aureus* ATCC 29213, and *E. faecalis* ATCC 29212, as well as against two Gram-negative reference strains *E. coli* ATCC 25922 and *P. aeruginosa* ATCC 27853. The results showed that only the dissymmetric compound **132** exhibits an important growth inhibition against *S. aureus* ATCC 25923, with an inhibition diameter of 31.5 mm.

**Figure 11:**
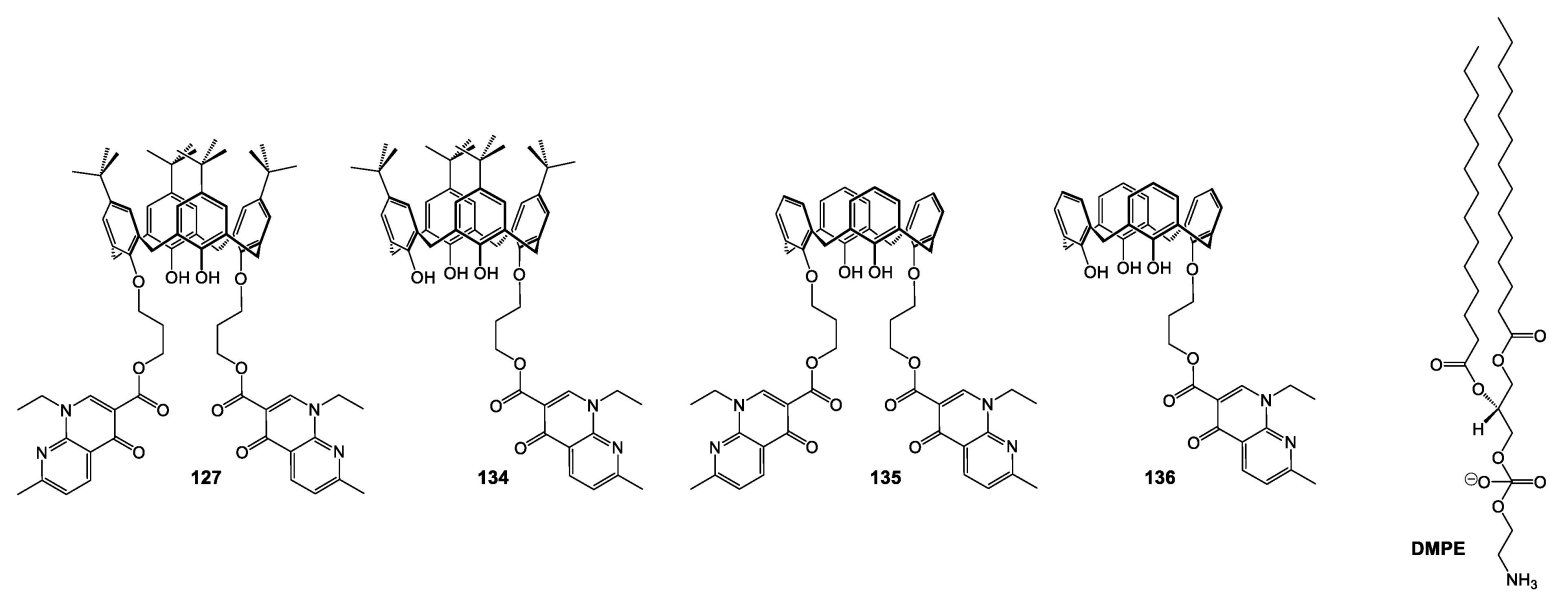
Schematic structures of the nalidixate podands **127**, **134**, **135**, **136**, and 1,2-dimyristoyl-sn-glycero-3-phosphoethanolamine (DMPE) [154].

To confirm the possible prodrug behavior of these compounds, Regnouf-de-Vains et al. reinvestigated the amphiphilic calixarenes bearing one or two nalidixic moieties attached as esters to the lower rim of the calixarene platform via a propyl linker, and H- or *tert*-butyl groups in the *para*-position at the upper rim [154]. The previously described compounds **127** [150] and **134** [151] were obtained in a better yield from bromopropylnalidixate in basic conditions (K_2_CO_3_ and NaHCO_3_ respectively) with KI as catalyst. The same conditions applied to detertiobutylated calix[4]arene gave the bis- and mono-nalidixate **118** and **119** (Figure 11).

The monolayers (monomolecular film) of amphiphilic calixarenes **127**, **134**, **135**, and **136** have been studied for the first time at the air–water interface on a Langmuir balance, showing a better stability of monolayers for the *para-tert*-butylated derivatives **127** and **134**. The films formed with derivatives **135** and **136** cannot be used in hydrolysis studies, as they are not stable enough. For a second time, the study at the air-carbonate buffer (pH 10) interface for **127** and **134** confirmed the initial hypothesis with a nalidixate salt release (HPLC monitoring).

The same nalidixic calixarenes **127** and **134** were studied upon interaction with model bacterial membranes via molecular dynamics simulation, as well as surface pressure, surface potential, or Brewster angle microscopy, for example, [155]. The results obtained show that mixed films formed with DMPE and calixarenes **127** or **134** have a more liquid-like character (more marked for **134**) compared with pure DMPE, and that interaction between the calixarene derivatives and DMPE occurs via the phosphate and carbonyl groups present in the lipid. Thus, diffusion of **134** in the monolayer would be easier compared with **127**, and this one can be useful for developing new calixarene based drug carriers.

**Scheme 14:**
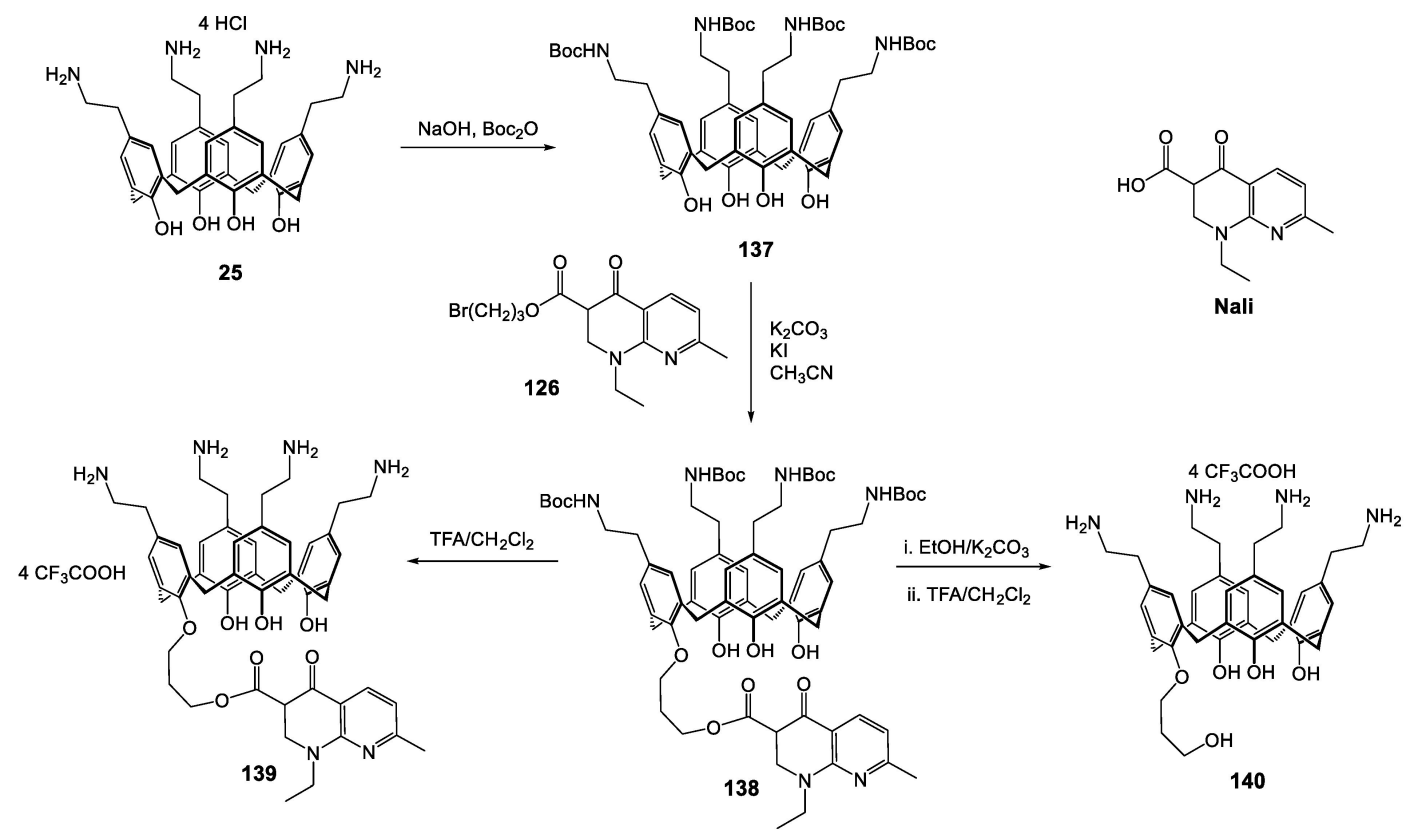
Synthetic pathway to podands **139** and **140** [156].

Designed as hydrophobic compounds, these structures are not really easy for in vitro standard evaluation as antimicrobial agents. Thus, in 2019, Massimba Dibama and co-authors developed a synthetic strategy leading to a water-soluble analogue of the previous compounds [156]. They described the synthesis of a prodrug-like structure that involves one nalidixic acid moiety tethered via a propyl linker to the lower rim of the *tetra*-*para*-aminoethylcalix[4]arene. Obtained according to Gutsche’s procedure [64], the *tetra-para*-aminoethylcalix[4]arene **25** was protected as Boc-derivative (**137** Scheme 14), and the introduction of bromopropylnalidixate **126** was performed under basic conditions in the presence of a catalytic amount of KI. Then the mononalidixic podand **138** was unprotected in TFA conditions to give the expected compound **139** (Scheme 14).

The antibacterial activities (MIC) of prodrug **139**, of its two expected metabolites nalidixic acid **Nali**, and 3-hydroxypropyl calix[4]arene **140** (Scheme 12) were evaluated in liquid phase against various Gram-negative (*E. coli* ATCC 25922, *P. aeruginosa* ATCC 27853) and Gram-positive (*S. aureus* ATCC 25923 and ATCC 29213, and *E. faecalis* ATCC 29212) bacteria. The calixarene species **139** displays MIC values of 35 µg/mL for *E. coli*, 70 µg/mL for the two *S. aureus* strains, and 140 µg/mL for *E. faecalis* and *P. aeruginosa*, while the mono-alcohol **140** needs from 2 (*E. faecalis*, *P. aeruginosa*) to 4 (*E. coli*, *S. aureus*) times higher concentrations, in ranges considered as non-active. **Nali** exhibits a MIC close to 32 µg/mL in all strains. The authors concluded that even with the ideal hypothesis of 100% hydrolysis, the MIC values obtained for the mononalidixate **139** are not consistent with the simple release of nalidixic acid, leading to a potential specific activity of **139** or an additive or a multiplicative effect between **139** or **140** and **Nali**.

On the same principle, Pur and Dilmaghani published, in 2014, the synthesis and biological evaluation of penicillins V and X clustered by a calixarene scaffold (named calixpenams) [157]. In this work, calixarene is not used as an active principle carrier; it becomes an integral part of it. They used the phenol moieties of calixarene to acylate the amino group of 6-aminopenicillanic acid (6-APA) and conduct the formation of cluster (cyclotetramer) of penicillin X (6-APA grafted on the upper rim) or penicillin V (6-APA grafted on the lower rim).

**Scheme 15:**
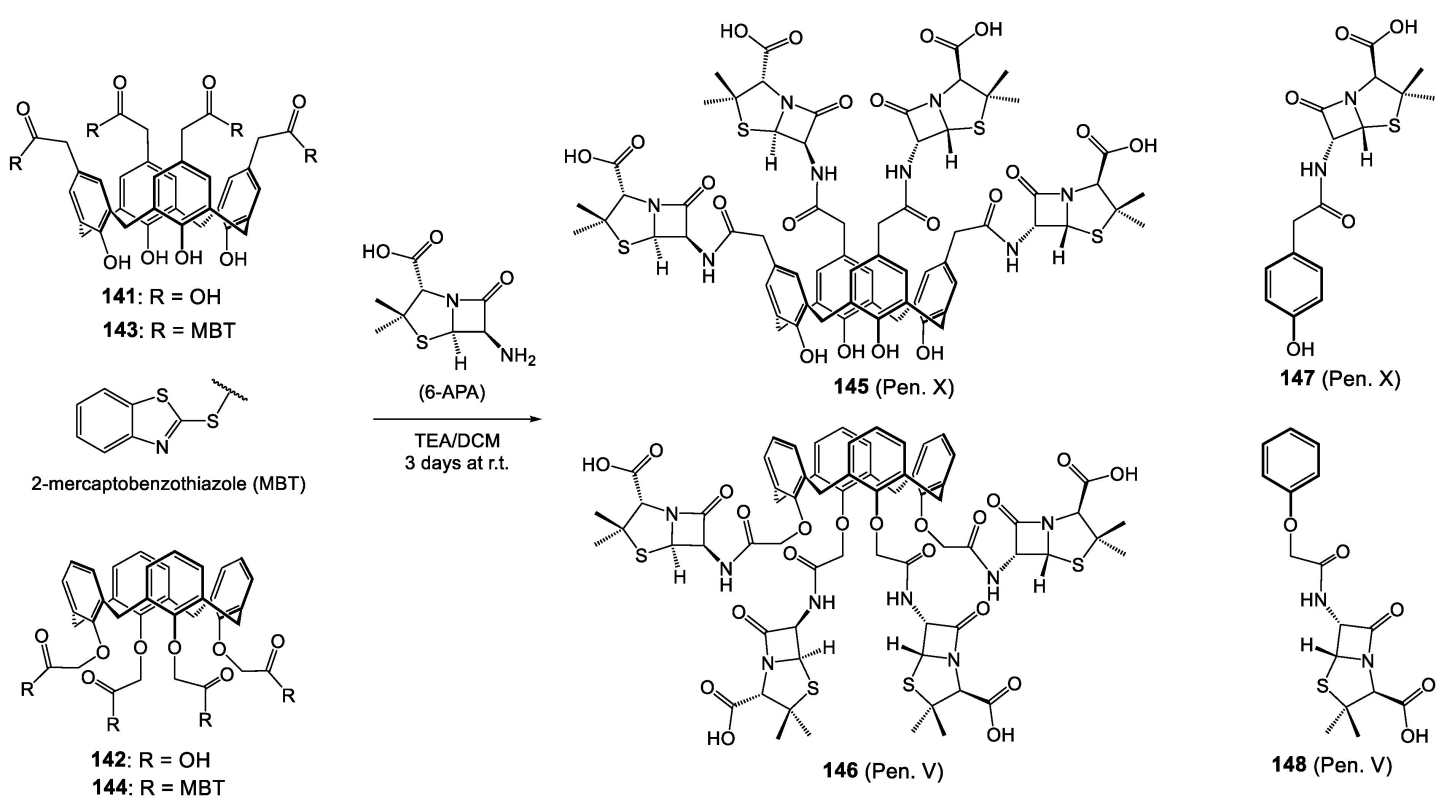
Synthetic pathway of calixpenams **145** (Pen. X), **146** (Pen. V) and their corresponding single penams **147** (Pen. X) and **148** (Pen. V) [157].

Previously prepared according the method of Gutsche et al. and McKuvey et al., respectively, the calixarenes **141** and **142** react in soft conditions via an active thioester formation process that involves the use of 2,2′dibenzothiazole disulfide (DBTDS) as carboxylic acid activator (**143** and **144** Scheme 15). Then the aminolysis reaction occurs in the presence of 6-APA and trimethylamine in CH_2_Cl_2_ to give the corresponding calixpenams **145** (58%) and **146** (49%), tetramers of penicillin X or penicillin V, respectively.

**Scheme 16:**
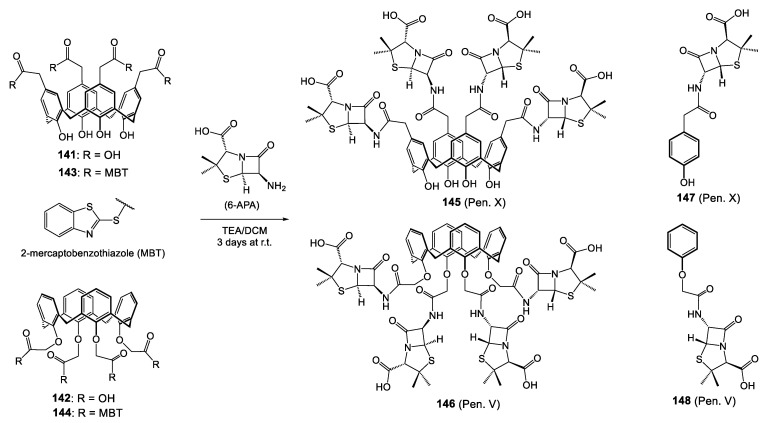
Synthetic pathway of calixcephems **151** (Ceph. X) and **152** (Ceph. V) and their corresponding single cephems **153** (Ceph. X) and **154** (Ceph. V) [158]. BSU: *N*,*N*′-bis(trimethylsilyl)urea, Py: pyridine.

For completeness, the authors conversed the calixpenams **145** and **146** to the corresponding calixcephems **151** and **152**, respectively (Scheme 16) [158]. This conversion was first through oxidation of sulfur atoms of penam moieties in the presence of peracetic acid (**149** and **150** Scheme 16), followed by ring expansion employing the *N*,*N*′-bis(trimethylsilyl)urea (BSU) and pyridine-hydrogen bromide (Py-HBr) to produce calixcephens **151** (Ceph. X) and **152** (Ceph. V) Scheme 16.

The antibacterial effect was initially measured against three Gram-positive non-penicillinase-producing strains of *Streptococcus* including *S. pyogenes* ATCC 19615, *S. agalactiae* ATCC 12386, and *Streptococcus pneumoniae* ATCC 49619 (Table 10) [157]. The impact of cyclotetramerization was demonstrated by a noteworthy increase in activity on all the strains of penicillin cyclotetramer (**145** and **146**) compared to the monomers **147** and **148**, respectively, for example, 0.002 mg/mL against *S. pyogenes* for **145** (Pen. X) and 0.012 mg/mL for its constitutive monomer **147** (Pen. X). It appears globally for both penams (X and V) a ratio of 6 between the calixarene’s MIC and the monomer’s MIC in favor of macrocycle. The authors attributed the slight difference of activity between **145** (Pen. X) (more active) and **146** (Pen. V) to a larger contact surface with the bacterial membrane offered by the upper rim of calixarene in the first case compared with that of the lower rim in the second.

In the second evaluation [158], the antibacterial susceptibility testing was assessed against five *S. aureus* strains: two β-lactamase-producing methicillin-resistant (MRSA) strains ATCC 43300 and ATCC 33591 and three methicillin-sensitive (MSSA) strains, including one non-β-lactamase producing strain ATCC 25923 and two β-lactamase-producing strains ATCC 29213 and 11632 (penicillin-resistant strain). The results also showed a great activity of calixarene structures versus the monomers. Calixcephens were about 10-fold more active than the corresponding monomer (1.550 µg/mL for **151** (Ceph. X) versus 16.325 µg/mL for constitutive monomer **153** (Ceph. X) on ATCC 3359). On the other hand, the comparison in the calixarene family demonstrates a considerable gain of activity for the calixcephems: 0.925, 1.225, and 1.635 µg/mL, respectively, on three β-lactamase-producing strains ATCC 11632, 43300 and 33591 versus the calixpenams >64 µg/mL for the same strains. These last results are probably due, according to the authors, to the fact that compounds including cephalosporanic acid cores have greater activity against β-lactamase-producing bacterial strains in comparison to compounds exhibiting penicillanic acid cores.

**Scheme 17:**
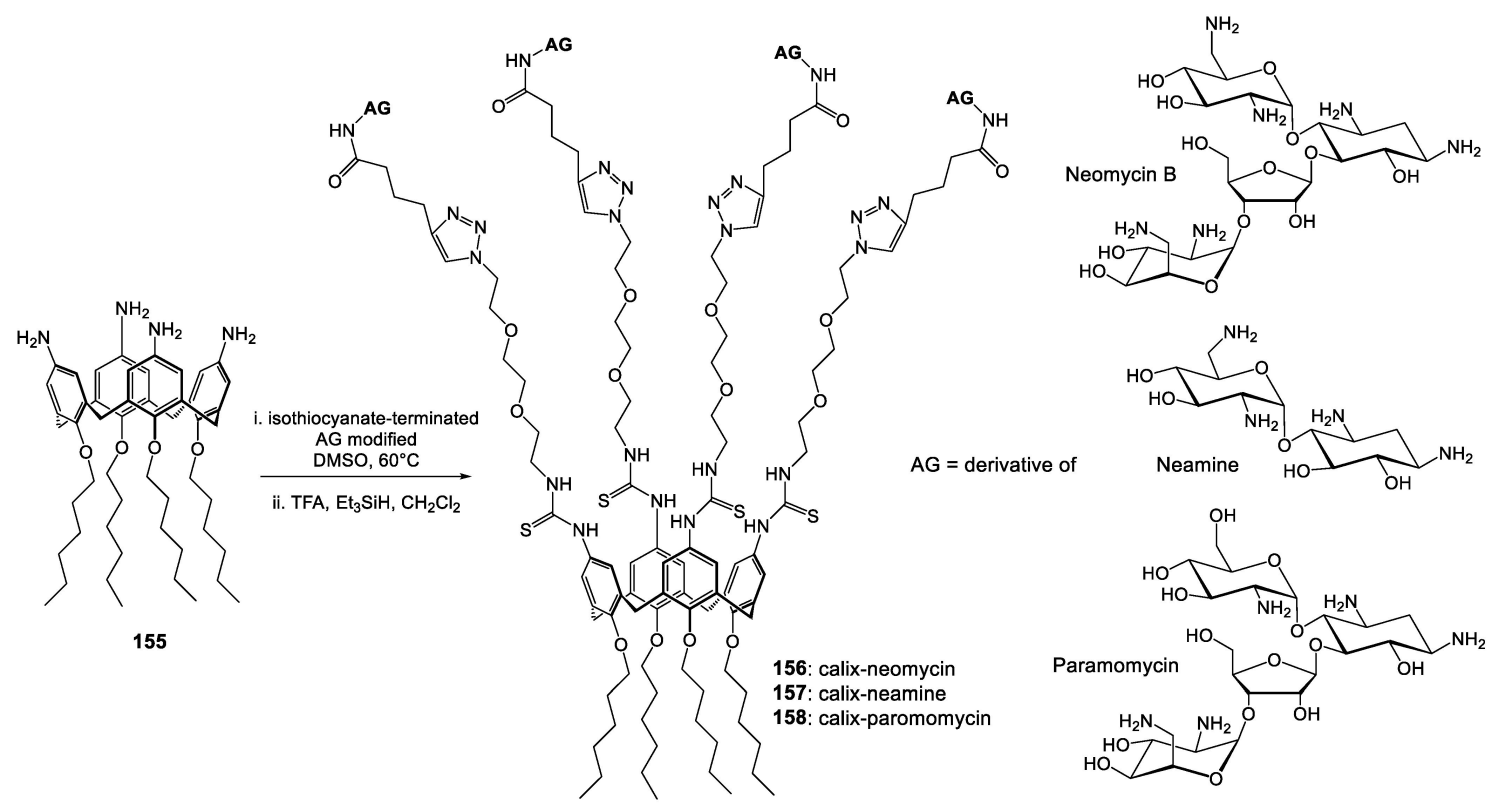
Synthetic pathway to calixarene-derivative- aminoglycoside-based **156**, **157**, and **158** [162].

Nowadays, many advances have been made in the search for new agents for nucleic acid (NA) delivery, notably through the use of pre-constituted building blocks and more particularly through the calixarene platform, due to its three-dimensional architecture and its possible multifunctionality increasing the interaction with NAs, as illustrated by the important work carried out by the Ungaro group’s examples [159,160,161]. Candiani and co-workers use this working basis for the development of new multivalent calixarenes and the study of their transfection efficiency [162]. The high affinity of aminoglycosides (AGs) for NAs, associated with their known antibacterial capabilities encourage the authors to cluster some AG derivatives on a calixarenic platform with the aim to obtain a new class of multivalent gene delivery vectors with inherent antibacterial properties (Scheme 17).

The starting material, the *para*-*tetra*-amino-*tetra*-hexyloxycalix[4]arene **155**, reacts with the isothiocyanate-functionalized aminoglycosides (AGs) derived from neomycin B, neamine and paromomycin in DMSO medium, giving rise to the formation of calix-AG derivatives **156**, **157**, and **158**, respectively (Scheme 17). If these three derivatives did induce very effective DNA complexation, they also have some antibacterial properties. These last were evaluated against Gram-negative *E. coli* and Gram-positive *Staphylococcus lutea* bacteria.

The starting calixarene **155** is ineffective, and the introduction of AGs results in the development of an antibacterial effect. In solution, derivatives **1156** and **1158** seem to be the most active and effectively inhibit the growth of *E. coli* compared with the inactive derivative **157**. For example, the paromomycin derivative **158** has a MIC_90_ around 28.2 µg/mL. However, it turns out that the calixarene-AG derivatives are still slightly less active than the free AGs used as references (neomycin and paromomycin show a MIC_90_ at 4 µg/mL). Surprisingly, the calixarene neamine derivative **157** is inactive, while its free AG displayed a MIC_90_ at 32 µg/mL. The authors attribute this lack of activity to the conjugation to the calixarenic skeleton, which, in this particular case, would produce an antagonistic effect to neighboring AGs. On the other hand, the same derivatives **156** to **158** displayed a weak antibacterial effect on Gram-positive strains.

### 7.3. Complexes

The ion complexation by calixarene derivatives has been the subject of numerous studies and is widely used in the separation and extraction of metal ions, sensors material or catalysts. They are good carriers for cations due to their cyclic structure with different sizes of cavities and the many possibilities of functionalization. These functional arms, endowed of electron donating groups, coupled to the organizational capacity of calixarene, allow generating a conducive environment to the ion’s reception.

**Scheme 18:**
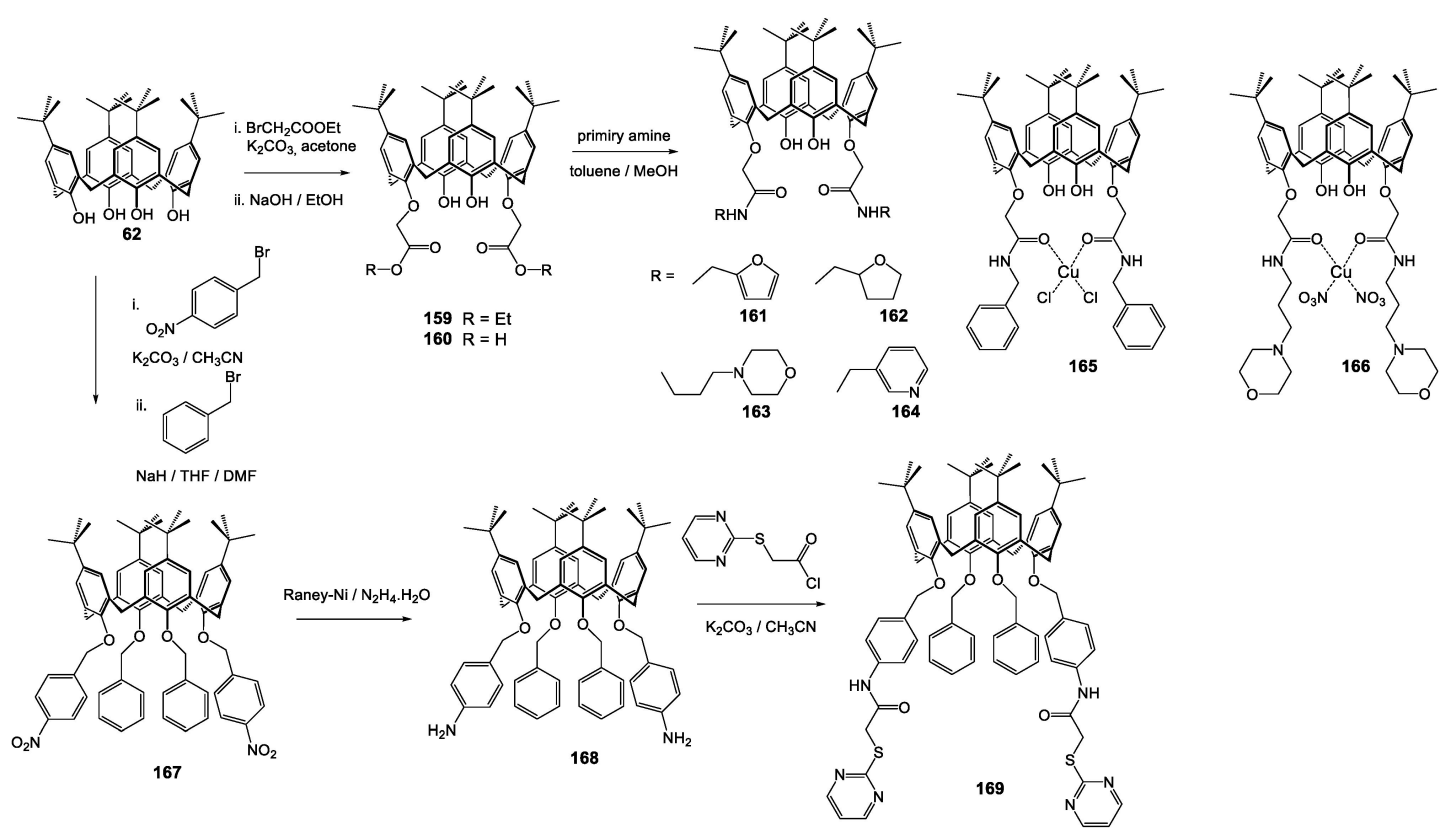
Synthetic route for obtaining ligand **161**, **162**, **163**, **165**, and **169** [163,164].

Through their research about DNA cleavage activity, Özkan et al. published, in 2015, a synthesis of the antimicrobial and DNA cleavage activities of *para*-*tert*-butylcalix[4]arene diamide derivatives and some of their copper(II)complexes [163]. After synthesising the diethyl-ester derivative **159** of *para*-*tert*-butylcalix[4]arene **62** (Scheme 18), hydrolysis process affords the di-acid compound **160**, which undergoes the introduction of amines to give the diamide derivatives **161** to **164** (Scheme 18). The capacities of complexation were carried out against some transition metal ions [Cu(II), Co(II), Cd(II), Zn(II), Ni(II), Mn(II), Pb(II)] and the best results are in favor of calixarene **163** and **164** for capture of Cu(II) giving Cu(II) complexes **165** and **166** (copper(II) chloride and copper(II) nitrate, respectively). These last two complexes were studied with ligands **161** to **164** for their antibacterial activities against Gram-positive (*B. subtilis* ATCC 6633, *B. cereus* NRRI-B-3711, *E. faecalis* (clinic sample), *S. aureus* NTCT 8325) and Gram-negative bacteria (*E. coli* ATCC 25922, *E. coli* ATCC 35128, *P. aeruginosa* ATCC 27853, *Proteus vulgaris* ATCC 8427) (Table 11).

The result of DNA interaction showed that of all the compounds tested, calixarene derivatives (**159**–**164**) and metal complexes (**165**, **166**) were able to cause damage to the plasmid DNA, and that copper(II) complexes are very effective for DNA cleavage. In parallel, the results of antibacterial susceptibility are less striking. The complexes are not active on the strains tested. The other structures **160**, **162**, **163**, and **164** showed no activity (MIC > 625 mg/mL); only the bi-ester intermediate **159** and the bis-furane derivative **161** give an inhibition of bacteria cell growth. *B. cereus, E. faecalis,* and *S. aureus E. coli (*ATCC 35128) are sensitive to compound **161** (MIC values 39, 39, 39, and 78 µg/mL, respectively). Surprisingly, it appears that compound **159** is the most active of the series and exhibits an activity on almost all strains, with MICs at 78 µg/mL on *B. subtilis*, 39 µg/mL on *B. cereus* and two strains of *E. coli*, and 156 µg/mL for *E. faecalis*, *S. aureus*, and *P. vulgaris*. The authors do not expose a reason for the activities detected for these two structures. They consider the non-activity of the copper(II) complexes by the largest molecular structures compared to compound **161**, for example, preventing the passage thought the cell bacterial membrane.

Based on a similar structure, Akkuş et al. published, the same year, a study on selective extraction of toxic heavy metals by a pyrimidylthioamide calixarene derivative and the corresponding antibacterial activities [164]. *Para*-*tert*-butylcalix[4]arene **62** was grafted in the *1,3-alternate* position via ether bond with benzyl moieties, then on the other phenolic positions by *para*-nitrobenzyl groups, in the presence of NaH as base (Scheme 18). The obtained compound **167** is placed in the presence of Raney-Ni, and the reduced derivative **168** which emerges reacts with 2-(pyrimidin-2-ylthio)acetyl chloride to afford the pyrimidylthioamide calixarene derivative **169** (Scheme 18). This last is a good extractant and has a good affinity for Hg and Pb ions with respect to the intermediates **167** and **168**. These mercury(II) or lead(II) complexes were not evaluated in antibacterial tests. On the other hand, the ligand **169** was tested for its antibacterial effect against the *E. coli* ATCC 25922, *K. pneumoniae*, *P. aeruginosa*, *S. aureus* NRRL-B 767, *Salmonella typhimurium*, *B. cereus* ATCC 11778, and *B. subtilis* NRS 744 bacterial strains. Measured by inhibition zones on an impregnated disc (5 mm) with 10 μg of drugs, an activity emerges against S. *typhimurium, B. cereus, B. subtilis, and S. aureus* (inhibition zone at 10, 10, 11, and 11 respectively) compared to 15, 8, 8, and 30 mm of inhibition obtained with Ceftriaxone used as reference. This activity of pyrimidylthioamide calixarene is associated by the authors with the presence of nitrogen and oxygen donor atoms and amide groups, which might have an activity against the enzyme production, in agreement with the literature [165].

**Scheme 19:**
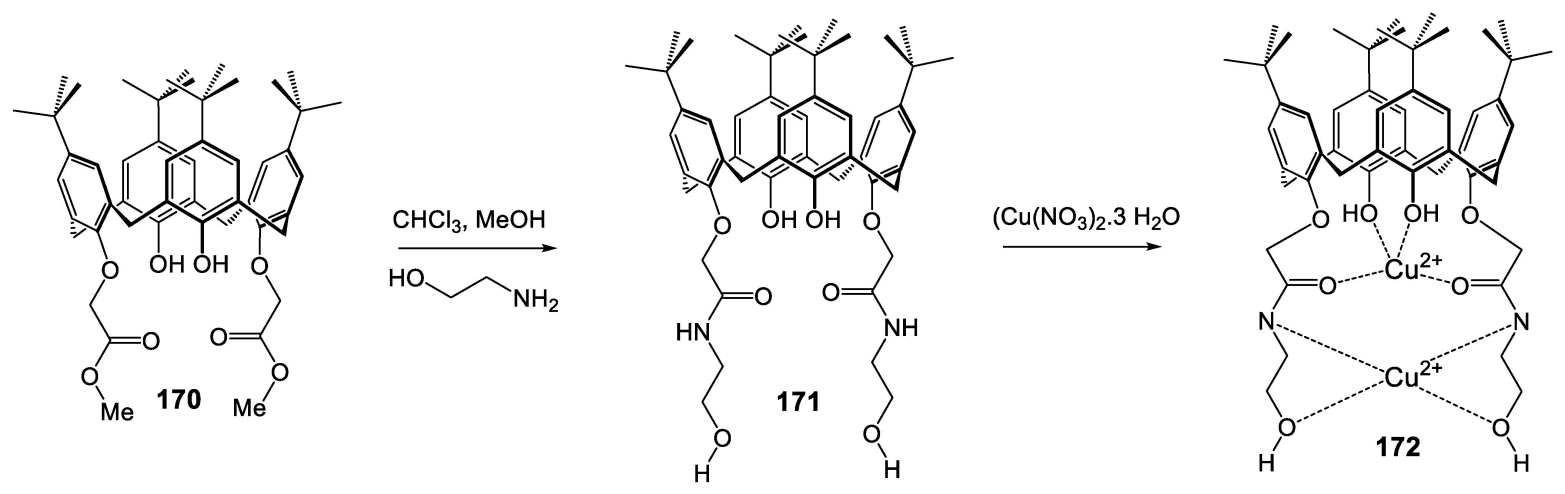
Synthetic pathway of binuclear copper complex **172** [166].

On the same principle, Memon et al. have synthesized a diamide derivative of *para*-*tert*-butylcalix[4]arene and its metal complex, and exhibit the antibacterial activities [166]. The *1,3*-*alternate* bis-amide ligand **171** was obtained by action of ethanolamine and methanol on the intermediate bis-ester **170**; then, the copper-complex **172** was formed via addition of stoichiometric amount of cuprique nitrate salt (Scheme 19).

Evaluation of bacterial growth was carried out Gram-positive *Staphylococcus albus* and Gram-negative *E. coli* ATCC strains with inhibition zone and MIC determinations on the ligand **171** and its copper complex **172** (Table 12).

The results show that these two calix[4]arene derivatives are very active against two bacteria strains tested. The organosoluble ligand **171** is active up to 3 µg/mL, while its binuclear Cu(II)-complex **172** generates an antibacterial activity eight times greater, with an active concentration at 0.37 µg/mL. The authors explain this variation of activity in favor of the complex by the fact that the complexation of the two copper atoms by the ligand makes it possible to increase the lipophilic nature of the structure via a significant delocalization and a sharing of electrons between the copper ions and the donor atoms. A greater lipophilicity thus allows easier passage of the lipid membrane, the complexes being able to cause problems in the process of respiration by blocking protein synthesis and therefore bacterial growth.

**Scheme 20:**
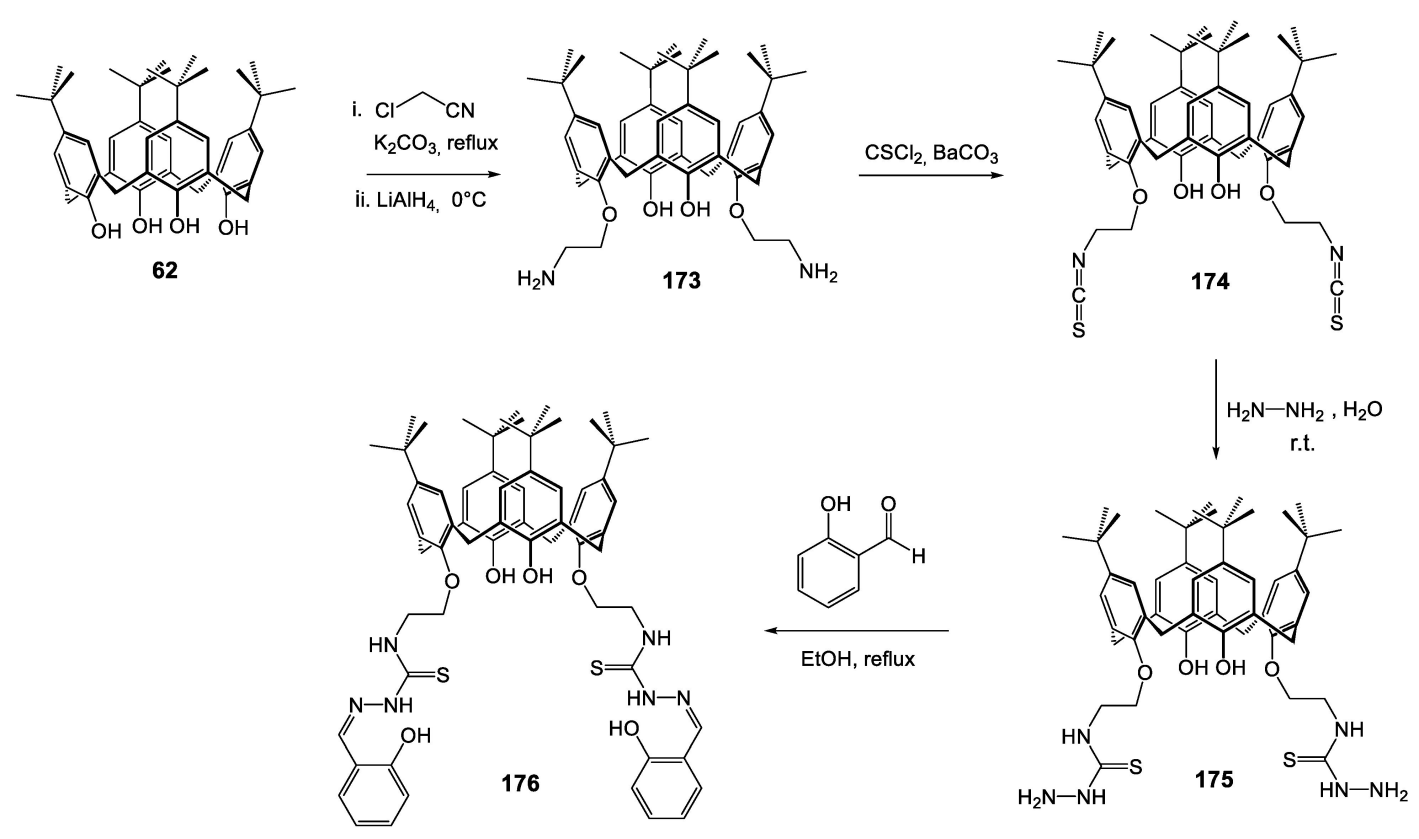
Synthetic route for obtaining of ligands **175** and **176** [167].

Multi-dentate chelating *para*-*tert*-butyl-calix[4]arene, functionalized at the 1,3-*alternate* positions of the lower rim with two thiosemicarbazide or thiosemicarbazone moieties, was reported by Bahojb Noruzi et al. in 2019 and 2020, respectively, with the aim to develop a new class of antimicrobial compound [167,168]. The thiosemicarbazide moieties and their thiosemicarbazone derivatives are known for their ability to coordinate with transition metals by bonding with sulfur and nitrogen atoms. They also studied, alone or in metal complex form, their biological properties, including antibacterial, antiviral, or antitumor activities. Weakly studied in literature, the calixarene-based thiosemicarbazone compounds could be an interesting candidate for antibacterial compounds. Therefore, the authors developed the ligand L following the synthetic way presented in Scheme 20. The *para*-*tert*-butylcalix[4]arene **62** functionalized in the *1,3-alternate* position by cyanoethyl arms followed by reduction step allows the bis-amino intermediate **173** formation. This last has been derived in isothiocyanate (compound **174**), then transformed in thiosemicarbazide using hydrazine (compound **175**). The expected thiosemicarbazone equivalent **176** was obtained by treatment of **175** with salicylaldehyde.

Eight divalent metal complexes were obtained by bringing **175** or **176** into contact with one equivalent of cobalt (II), nickel (II), copper (II), or zinc (II) salts to afford the metal derivatives **175-Co**, **175-Ni**, **175-Cu**, **175-Zn**, **176-Co**, **176-Ni**, **176-Cu,** and **176-Zn**. All of the synthesized compounds were evaluated with regard to their antibacterial capacity against Gram-positive bacteria (*S. aureus* and *B. subtilis*) and Gram-negative bacteria (*E. coli* and *P. aeruginosa*) and the MIC values are presented in Table 13.

The first observation is the fact that the thiosemicarbazide ligand **175** is almost inactive against the tested strains, while the thiosemicarbazone **176** analogue exhibits a stronger antibacterial effect with an increase in MIC values of 2-fold and 8-fold against *P. aeruginosa* and *E. coli*, respectively, i.e., 31.25 µg/mL on these strains. No decrease in bacterial growth is observed for *S. aureus*. In the case of **175**, all of the metal derivatives show an enhancement of the antibacterial activity against Gram-negative bacteria (except for the Co(II) and Cu(II) derivatives against *P. aeruginosa*), with a more significant improvement for the Ni(II) and Zn(II) complexes. In parallel, the **176** complexes are all very active on the all strains tested in the study, with MICs of 31.25 µg/mL, and are thus found to be much more interesting in terms of activity than their thiosemicabazide-based analogues.

## 8. Outlook

Several important points emerge from this review. First, it is undeniable that the organizational capacity of the calixarene plays a pivotal role in the antimicrobial activities observed. Second, numerous works propose decorating the calixarene structure with positively charged functions (e.g., ammonium, guanidinium, imidazolium…) to most likely target the bacterial cell wall. Third, the antibacterial activities measured are very promising: broad spectrum activity, against both wild-type bacteria or antibiotic-resistant bacteria. Fourth, even if the majority of the reported work is based on calixarenes with conical conformation, others are beginning to focus on other conformations, and the authors reach identical conclusions: significant differences in terms of activity in function of conformation. Thus, we can admit that the stereochemical orientation of the functional groups present on the surface of the calixarenic skeleton plays on the antibacterial activity. Fifth, several studies also show that the multivalence offered by calixarenes seems very favorable to the development of prodrugs (e.g., modification of bioavailability, cluster effect, hydrolyzable link, etc.). Finally, all of these studies should lead us to more in-depth research on structure-activity relationships and the design of new functionalized calixarenes with enhanced antibacterial properties.

Nonetheless, the use of calix[*n*]arenes in the therapeutic fields, particularly for infectious diseases, is still recent, which, despite their numerous advantages, results (still) in an undervaluation of their biological properties, or even a lack of interest. However, the almost unlimited possibilities for functionalization of these structures, associated with their multivalence and their potential for spatial organization, offer a very wide range of possibilities for the development of promising candidates usable in different medical fields, and more precisely, bacteriology. However, if the antibacterial activities are very promising, the compounds currently studied will still require more in-depth studies to possibly become potential drug candidates.

## 9. Conclusions

Calixarenes have already come a long way since A. von Baeyer in 1872 and have found their place in many fields of study and applications. Thanks to their great flexibility (functionalization and spatial organization), the Man of Art knew how to modulate them in order to confer remarkable activities. If we only consider the discrete calixarene structures, the subject of this review, they have found a place in the first stage of the therapeutic field, and more particularly, the search of new antibacterial agents. The crucial lack of new antibiotics associated with the alarming increase in bacterial resistance requires a major effort in the search for new therapeutic strategies, and it appears that the calixarene structure finds its place there. The researchers have thus designed many structures carrying intrinsic activity, activity linked to the possibility of releasing active principles or to the formation of metal ion complexes. From average to very good, these activities make it possible to determine structure–activity relationships and to direct researchers towards structures with higher potential. For example, many works report the development of cationic calixarenes with an original mechanism of action based on electrostatic interactions with the bacterial cell wall. The very interesting results highlighted could lead to improvement in future work. The exceptional modularity (regioselective polyfunctionalization and multiple conformations) of the calixarene structure can still allow researchers to shape their structures in order to further increase the affinities with the targets of action sought, to broaden the spectrum of action, or, on the contrary, to target more specific strains.

## Figures and Tables

**Table 1:** Antibacterial activities of compounds **7** to **14**; zone of inhibition corresponds to the total diameter zone [114].

		Compounds							
Bacterial Strains		7	8	9	10	11	12	13	14
*E. coli*	10 mg/mL	-	++++	-	-	-	-	-	-
	5 mg/mL	-	++++	-	-	-	-	-	-
*S. aureus*	10 mg/mL	-	++++	++++	-	+++	++	++++	-
	5 mg/mL	-	++++	++++	-	++	+	++++	-
	1 mg/mL	-	++	+++	-	++	-	+	-
*B. subtilis*	10 mg/mL	+++	-	++++	++++	-	-	++++	-
	5 mg/mL	+	-	+++	-	-	-	++++	-
	1 mg/mL	-	-	++	-	-	-	++	-
*K. pneumoniae*	10 mg/mL	++++	-	-	-	+++	++++	-	++++
	5 mg/mL	+++	-	-	-	++	+++	-	+++
*P. aeruginosa*	10 mg/mL	++++	-	-	-	+++	-	-	-
	5 mg/mL	+++	-	-	-	++	-	-	-

Zone of inhibition: -: none; +: 6 mm; ++: 7 mm; +++: 10 mm; ++++: 14 mm.

**Table 2:** MICs (Minimum Inhibitory Concentrations in µg/mL) of peptidocalixarene and vancomycin against several bacterial strains of interest [115].

	Compounds				
Reference Strains	Vancomycin	17	18	19	20
*S. aureus* 663	2	64	8	8	32
*S. aureus* 853	2	>64	16	8	32
*S. aureus* 1131	2	64	4	4	32
*S. epidermidis*	2	32	4	8	32
*B. cereus*	2	nt	16	nt	nt

*S. aureus* 663 = penicillin-sensitive, *S. aureus* 853 = penicillin-resistant, *S. aureus* 1131 = methicillin-resistant, nt = not tested.

**Table 3:** MICs (in µg/mL) of **28** and its constitutive monomer **29** against ATCC reference strains and clinical isolates, respectively. HX (hexamidine) and CHX (chlorhexidine) were used as reference antibacterial agent [72].

	Compounds			
Strains	28	29	HX	CHX
Reference strains				
*E. coli* ATCC 25922	4	512	8	0.25
*P. aeruginosa* ATCC 27853	32	>512	32	8
*S. aureus* ATCC 25923	8	512	4	0.5
*S. aureus* ATCC 29213	8	512	<1	0.5
*E. faecalis* ATCC 29212	32	>512	2	2
Clinical isolates				
Penicillinase-producing *E. coli*	2	512	8	nd
MRSA (mecA gene)	8	512	2	nd
*E. faecium* (vanB gene)	8	>512	2	nd
*E. faecalis* (vanA gene)	64	>512	4	nd
*P. aeruginosa* (overexpression of efflux pumps)	64	>512	64	nd

nd: not determined.

**Table 4:** CI_50_ and MICs (in µg/mL) of **28** and its bis-heterocycle derivatives **36** to **38** on MRC-5 cells and against reference bacterial strains, respectively [128].

	Compounds			
	28	36	37	38
CI_50_	>256	16–32	>128	64–128
Reference strains				
*E. coli* ATCC 25922	4	16	32	16
*P. aeruginosa* ATCC 27853	32	16	32	16
*S. aureus* ATCC 25923	8	16	8	8
*S. aureus* ATCC 29213	8	16	8	16
*E. faecalis* ATCC 29212	32	16	8	8

**Table 5:** MICs (in µg/mL) of **25**, **28**, **34**, **37**, **38** against reference strains H_37_Rv and MYC5165. Isoniazid (INH) was used as reference [132].

	Compounds					
	25	28	34	37	38	INH
Strains						
*M. tuberculosis* H37Rv	2.58	1.0	1.34	1.51	2.69	0.08
*M. tuberculosis* MYC5165	2.6	1.0	2.7	0.75	0.17	1.7

**Table 6:** MICs (in µg/mL) of cationic calixarenes modified with methylimidazolium (**70**, **71**), choline (**72**, **73**) and ethylenediamine (**74**, **75**) groups against ATCC reference strains [135].

	Compounds								
Reference Strains	70 (prop)	72 (prop)	74 (prop)	71 (oct)	73 (oct)	75 (oct)	18/19 ^a^	28 ^a^	61 ^a^
*S. aureus* ATCC 6538	<10	<10	<10	>1000	>1000	>1000	8	8	16
*P. aeruginosa* ATCC 9027	50	1000	50	>1000	500	>1000	nd ^b^	32	nd ^b^
*K. pneumoniae* ATCC 10031	<10	100	500	500	>1000	>1000	nd ^b^	8	4
*E. coli* ATCC 25922	<10	100	500	1000	500	>1000	3.2	4	4

^a^ no data for these specific strains, MICs for species is shown. ^b^ nd: no data available.

**Table 7:** MIC values (in µg/mL) of azo-compounds **81** to **86** against several bacterial strains [133].

	Compounds					
Bacterial Strains	81	82	83	84	85	86
*S. aureus*	7.8	3.9	7.8	>125	>125	>125
MRSA	15.6	0.97	7.8	>125	31.25	>125
*B. subtilis*	15.6	0.97	0.97	>125	125	>125
*S. epidermidis*	7.8	1.9	1.9	>125	62.5	>125
*E. faecalis*	31.25	7.8	7.8	>125	15.6	>125
*E. coli*	>125	>125	125	>125	>125	>125
*P. aeruginosa*	15.6	>125	62.5	>125	62.5	>125

**Table 8:** MICs (in µg/mL) of guanidinium derivatives **92**, **93**, **94**, **95**, and **96** against the various bacteria used in the study [103].

	Compounds				
Reference Strains	92 *cone*	93 *paco*	94 *1,2-alt*	95 *1,3-alt*	96
*E. coli* ATCC 25922	64	8	8	4	64
*P. aeruginosa* ATCC 27853	128	8	16	16	128
*S. aureus* ATCC 25923	32	2	4	<1	32
*S. aureus* ATCC 29213	32	4	8	8	32
*E. faecalis* ATCC 29212	8	4	8	8	128
*M. tuberculosis* H_37_Rv	78	38	39	5	10
*M. tuberculosis* MYC5165	9.5	2.4	3.8	1.2	37.2

Note: MICs of INH: 0.16 and 20 μg/mL against H_37_Rv- and INH-resistant MYC5165, respectively.

**Table 9:** MICs (in µg/mL) of ammonium derivatives **104** to **113** against the various bacteria used in the study [147].

		Compounds										
		104	109	108	113	105	110	106	111	107	112	CHX
	R=	Me		Bn		Bu		Oct		Dec		
Reference Strains		*cone*	*1,3-alt*	*cone*	*1,3-alt*	*cone*	*1,3-alt*	*cone*	*1,3-alt*	*cone*	*1,3-alt*	
*S. aureus* ATCC 29213		8	>64	4	4	1	16	64	1	32	4	2
*S. epidermis* ^a^		4	>64	2	4	1	16	64	1	8	4	1
*B. subtilis* 168		1	64	4	1	2	8	4	2	4	4	2
*E. coli* ATCC 25922		>64	>64	>64	32	>64	64	>64	8	>64	16	1
*K. pneumoniae* 1813		>64	16	>64	32	>64	1	8	32	4	32	2
*P. aeruginosa* ATCC 27853		>64	>64	>64	>64	>64	>64	>64	16	>64	64	4

^a^: clinical isolate.

**Table 10:** MICs (in µg/mL) (by broth microdilution) of calixpenams (**145** (Pen. X), **146** (Pen. V)), calixcephems (**151** (Ceph. X), **152** (Ceph. V)) and their corresponding single penams (**147** (Pen. X), **148** (Pen. V)) and cephems (**153** (Ceph. X), **154** (Ceph. V)), respectively [157].

	Compounds							
Reference Strains	145 (Pen. X)	146 (Pen. V)	147 (Pen. X)	148 (Pen. V)	151 (Ceph. X)	152 (Ceph. V)	153 (Ceph. X)	154 (Ceph. V)
*S. pyogenes* ATCC 19615	0.002	0.003	0.012	0.016	nt	nt	nt	nt
*S. agalactiae* ATCC 12386	0.004	0.006	0.025	0.032	nt	nt	nt	nt
*S. pneumoniae* ATCC 49619	0.022	0.024	0.125	0.125	nt	nt	nt	nt
*S. aureus* ATCC 25923	0.025	0.032	nt	nt	0.225	0.25	1.775	1.550
*S. aureus* ATCC 29213	0.24	0.256	nt	nt	0.992	1.012	5.325	5.150
*S. aureus* ATCC 11632	>64	>64	nt	nt	0.912	0.925	8.775	8.350
*S. aureus* ATCC 43300	>64	>64	nt	nt	1.150	1.225	11.775	10.950
*S. aureus* ATCC 33591	>64	>64	nt	nt	1.550	1.635	16.650	16.325

nt: not tested.

**Table 11:** MICs (in µg/mL) of calixarene derivatives **159** to **166** against ATCC reference strains [163].

	Compounds							
Reference Strains	159	160	161	162	163	164	165	166
*B. subtilis* ATCC 6633	78	5000	1250	1250	10,000	10,000	nd	nd
*B. cereus* NRRI-B-3711	39	10,000	39	2500	10,000	10,000	nd	nd
*E. faecalis (clinical isolate)*	156	10,000	39	1250	10,000	1250	nd	nd
*S. aureus* NTCT 8325	156	10,000	39	1250	10,000	625	nd	nd
*E. coli* ATCC 25922	39	10,000	625	10,000	10,.000	1250	nd	nd
*E. coli* ATCC 35128	39	5000	78	5000	5000	2500	nd	nd
*P. aeruginosa* ATCC 27853	1250	5000	5000	5000	5000	5000	nd	nd
*P. vulgaris* ATCC 8427	156	10,000	5000	5000	5000	2500	nd	nd

nd: No MICs determined in the range of tested concentrations (≥10,000 μg/mL).

**Table 12:** Zones of inhibition (in mm) and MICs (in μg/mL) of compound **171** and its Cu(II)-complex **172** [166].

	Compounds											
	171						172					
Conc. in μg/mL	6	3	1.5	0.75	0.37		6	3	1.5	0.75	0.37	
Reference Strains	Inhibition Zone					MIC	Inhibition Zone					MIC
*S. albus*	6	2	-	-	-	3	11	8	5	3	-	0.75
*E. coli*	8	3	-	-	-	3	12	10	7	5	2	0.37

**Table 13:** MICs (in µg/mL) of synthesized calix[4]arene-based ligands **175** and **176** as well as their metal complexes against bacterial reference strains [167].

	Compounds									
Reference Strains	Thiosemicarbazide					Thiosemicarbazone				
	175	175-Co	175-Ni	175-Cu	175-Zn	176	176-Co	176-Ni	176-Cu	176-Zn
*S. aureus* ATCC 29213	-	250	-	250	500	-	-	-	31.25	-
*B. subtilis* ATCC 6633	31.25	-	31.25	31.25	125	31.25	31.25	31.25	31.25	31.25
*E. coli* ATCC 25922	250	125	62.5	125	31.25	31.25	31.25	31.25	31.25	31.25
*P. aeruginosa* ATCC 27853	62.5	125	31.25	62.5	31.25	31.25	31.25	31.25	31.25	31.25

## Data Availability

Not applicable.
